# The SnRK2-APC/C^TE^ regulatory module mediates the antagonistic action of gibberellic acid and abscisic acid pathways

**DOI:** 10.1038/ncomms8981

**Published:** 2015-08-14

**Authors:** Qibing Lin, Fuqing Wu, Peike Sheng, Zhe Zhang, Xin Zhang, Xiuping Guo, Jiulin Wang, Zhijun Cheng, Jie Wang, Haiyang Wang, Jianmin Wan

**Affiliations:** 1 National Key Facility for Crop Gene Resources and Genetic Improvement, Institute of Crop Science, Chinese Academy of Agricultural Sciences, Beijing 100081, China; 2 National key Laboratory for Crop Genetics and Germplasm Enhancement, Jiangsu Plant Gene Engineering Research Center, Nanjing Agricultural University, Nanjing 210095, China

## Abstract

Abscisic acid (ABA) and gibberellic acid (GA) antagonistically regulate many developmental processes and responses to biotic or abiotic stresses in higher plants. However, the molecular mechanism underlying this antagonism is still poorly understood. Here, we show that loss-of-function mutation in rice Tiller Enhancer (TE), an activator of the APC/C^TE^ complex, causes hypersensitivity and hyposensitivity to ABA and GA, respectively. We find that TE physically interacts with ABA receptor OsPYL/RCARs and promotes their degradation by the proteasome. Genetic analysis also shows OsPYL/RCARs act downstream of TE in mediating ABA responses. Conversely, ABA inhibits APC/C^TE^ activity by phosphorylating TE through activating the SNF1-related protein kinases (SnRK2s), which may interrupt the interaction between TE and OsPYL/RCARs and subsequently stabilize OsPYL/RCARs. In contrast, GA can reduce the level of SnRK2s and may promote APC/C^TE^-mediated degradation of OsPYL/RCARs. Thus, we propose that the SnRK2-APC/C^TE^ regulatory module represents a regulatory hub underlying the antagonistic action of GA and ABA in plants.

## Abstract (web summary)

The hormones abscisic acid and gibberellins act antagonistically in plant development and stress responses. Here Lin *et al*. show that the rice Tiller Enhancer protein is required for gibberellin-induced degradation of abscisic acid signalling components, uncovering mechanistic insights into hormone signalling crosstalk.

## Graphical abstract

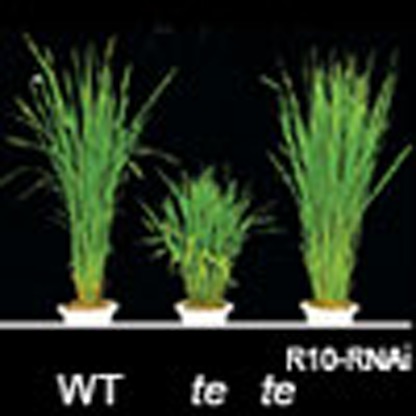

Abscisic acid (ABA) is a stress responsive phytohormone that inhibits seed germination and seedling growth to adapt to unfavourable environmental conditions while gibberellic acid (GA) is a major growth promoting phytohormone that promotes seed germination, seedling growth, flowering and leaf expansion^1^,^2^,^3^. Recent studies have portrayed a conceptual ABA signalling pathway in which ABA binds to its receptor PYL/PYR/RCARs, subsequently the PYL/PYR/RCAR-ABA complex binds to PP2C phosphatases that repress the SnRK2s, releasing the activated SnRK2s to phosphorylate downstream targets to activate ABA responses^4^,^5^,^6^,^7^,^8^,^9^,^10^,^11^. In the GA signalling pathway, the receptor GID1 and E3 ligase SCF^SLY1/GID2^ together promote the degradation of the DELLA repressor proteins in a GA-dependent manner to relieve their repression of GA action^12^,^13^,^14^. Although recent studies have shown that ABA can antagonize GA-promoted degradation of DELLA proteins^15^, the regulatory mechanism underlying the antagonism of GA on ABA signalling pathway remains largely unknown.

In a previous study, we reported that rice *Tiller Enhancer* (*TE*) encodes an activator of the APC/C^TE^ E3 ubiquitin ligase complex that acts to repress tillering (branching) by promoting the degradation of MOC1, a master regulator of plant architecture and tiller number^16^. In this study, we find that the loss-of-function *te* mutant displays increased sensitivity to ABA, but reduced sensitivity to GA. We show that APC/C^TE^ can repress the ABA signalling by mediating the degradation of ABA receptors. Conversely, ABA can inhibit the APC/C^TE^-mediated degradation of ABA receptors by interrupting the interaction between TE and ABA receptors through activating the SnRK2-mediated phosphorylation of TE. In contrast, GA can promote the APC/C^TE^-mediated degradation of ABA receptors by enhancing the interaction between TE and ABA receptors through reducing the protein levels of SnRK2s. Our results unravel a novel mechanism underlying the antagonistic action of GA on ABA signalling pathway.

## Results

### Contrary responses of *te* mutant to ABA and GA

**Figure 1:**
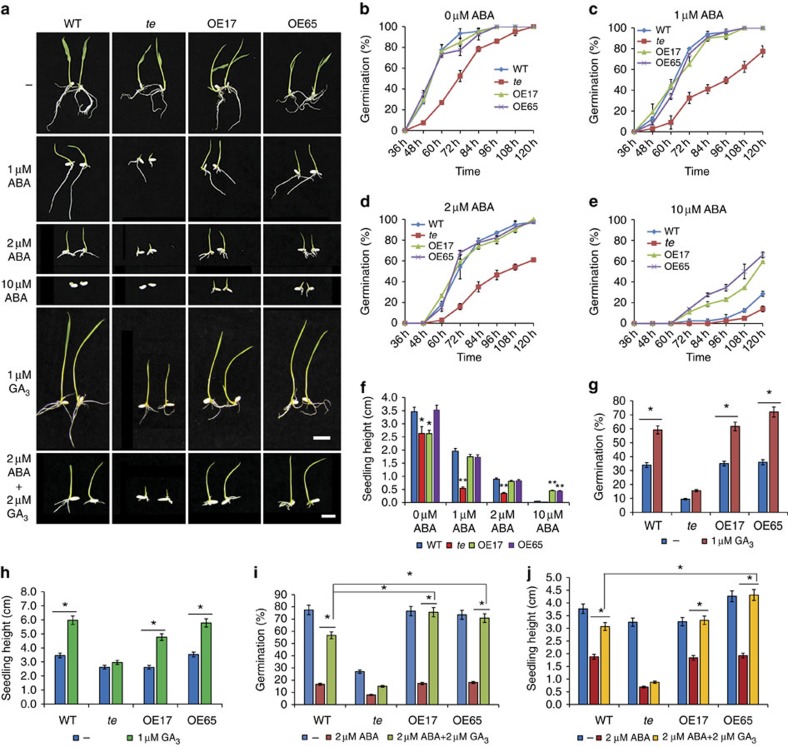
ABA and GA responses of *te* mutant and *TE* overexpression lines. (**a**) Seedling growth phenotypes of WT, *te* and *TE* overexpression lines (OE17 and OE65) treated with ABA, GA_3_ or both ABA and GA_3_. Photographs were taken on day 5. Scale bars, 1 cm. (**b**–**e**) Germination time courses on MS medium containing 0 μM ABA (**b**), 1 μM ABA (**c**), 2 μM ABA (**d**) or 10 μM ABA (**e**). (**f**) Seedling heights of WT, *te*, OE17 and OE65 grown for 5 days on MS containing 0 μM ABA, 1 μM ABA, 2 μM ABA or 10 μM ABA. The Student's *t*-test analysis indicated a significant difference compared with WT (*P<0.05, **P<0.01). (**g**) Germination rates of WT, *te*, OE17 and OE65 at 48 h on MS medium without GA_3_ or with 1 μM GA_3_. (**h**) Seedling heights of WT, *te*, OE17 and OE65 grown for 5 days on MS medium without GA_3_ or with 1 μM GA_3_. (**i**) Germination rates of WT, *te*, OE17 and OE65 at 60 h on MS medium containing no growth regulators, 2 μM ABA or both 2 μM ABA and 2 μM GA_3_. (**j**) Seedling heights of WT, *te*, OE17 and OE65 grown for 5 days on MS medium containing no growth regulators, 2 μM ABA or both 2 μM ABA and 2 μM GA_3_. The Student's *t*-test analysis indicated a significant difference (*P<0.05) in **g**–**j**. Values are means±s.d. in **f** and **j** (*n*=30 seedlings).

In addition to increased tillers, the *te* mutant displays a pleiotropic phenotype, including reduced height, twisted flag leaf and panicles, suggesting that TE regulates multiple developmental processes^16^. In this study, we found that compared with wild-type (WT) plants, *te* mutant also displayed delayed seed germination and seedling growth in the presence or absence of exogenously supplied ABA; in contrast, the *TE* overexpression lines (OE17 and OE65) displayed accelerated seed germination and seedling growth compared with WT when grown on Murashige and Skoog (MS) medium supplemented with 10 μM ABA (Fig. 1a–f), suggesting that TE may play a role in regulating ABA responses. Consistent with this notion, *te* mutants were also more tolerant to drought stress, compared with WT plants (Supplementary Fig. 1a,b). Quantitative reverse transcriptase–PCR (qRT–PCR) analyses showed that the expression of representative ABA-responsive genes *LEA3* and *RAB16A* was slightly increased in *te* plants, but the *LEA3*, *LIP9* and *RAB16A* mRNAs were clearly reduced in the OE17 plants (Supplementary Fig. 1c–e). ABA levels were not significantly different in *te*, OE17 and WT plants (Supplementary Fig. 1f). These results suggest that TE likely acts as a repressor of ABA signalling.

Conversely, we found that the *te* mutant displayed a reduced response to GA_3_ in seed germination and seedling growth, as well as GA-promoted α-amylase activity, compared with WT and the *TE* overexpression lines, OE17 and OE65 (Fig. 1a,g and h and Supplementary Fig. 2a). qRT–PCR assay showed that expression of the α-amylase gene *RAmy1A* was dramatically induced in OE17 plants (Supplementary Fig. 2b–d), indicating that overexpression of *TE* conferred a hypersensitivity to GA. Thus, *TE* appears to play a positive role in regulating GA signalling. Further assays showed that ABA treatment inhibited the germination and growth of *te* more significantly than WT, OE17 and OE65, and this inhibition was more effectively blocked by 2 μM GA_3_ in WT, OE17 and OE65 than in *te* mutant (Fig. 1a,i and j). These results suggest that TE is involved in mediating the antagonistic action of ABA and GA.

### APC/C^TE^ targets ABA receptors for degradation

**Figure 2:**
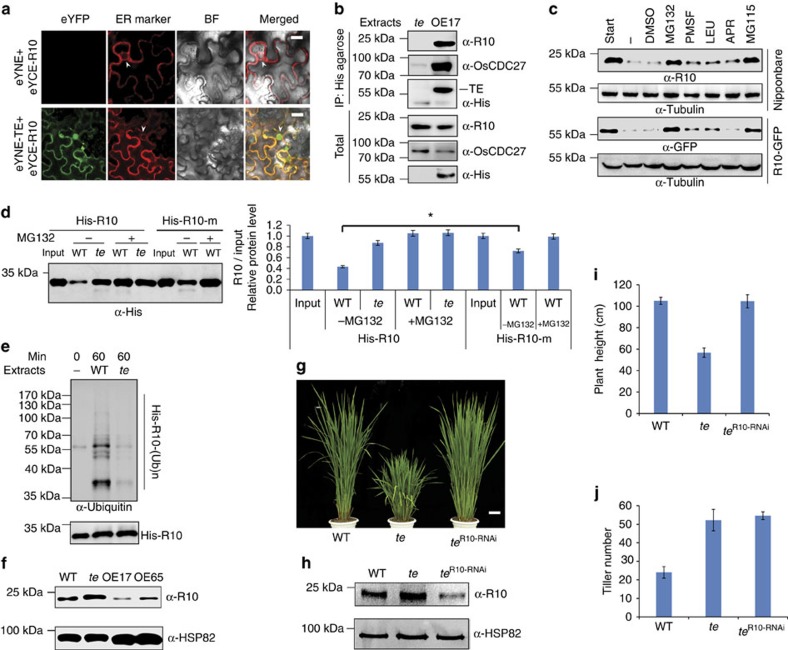
TE mediates the degradation of OsPYL/RCAR10 (R10). (**a**) BiFC analysis shows interaction between TE and R10 in *N. benthamiana* leaf epidermis cells. eYFP, eYFP fluorescence; ER marker, a fluorescent marker protein localized to the endoplasmic reticulum; BF, blight-field image. White arrowheads indicate the nuclear membrane. Scale bar, 50 μm. (**b**) Co-immunoprecipitation assay shows His-agarose simultaneously pulled down TE, OsCDC27 and R10 from the OE17, but not *te*, plant extracts by specifically binding the His epitope at the TE N-terminus. (**c**) Cell-free degradation assay shows that the degradation of R10 in WT (cv. Nipponbare) or R10-GFP in the *R10-GFP* overexpression line is effectively blocked by MG132 or MG115 but not by PMSF, LEU, APR or DMSO. (**d**) Cell-free degradation assay shows that the mutant His-R10-m protein with a mutated D-box (RLDL→ALDA) remains relatively stable compared with His-R10 WT protein in WT plant extracts. ‘Input' shows that roughly equal amounts of His-R10-m and His-R10 proteins were used. Left panel shows the representative western blot and the right panel shows the quantification analysis of relative R10/input protein levels corresponding to the left panel. Error bars represent the s.d. from triplicate experiments. Asterisks mark significant differences in relative R10/input protein levels between His-R10 and His-R10-m in WT plant extracts without MG132 treatment according to the Student's *t*-test (*P<0.05). (**e**) *In vitro* ubiquitination assay shows that His-R10 is polyubiquitinated more effectively by the WT than by the *te* plant extracts. (**f**) Western blot analysis shows that the levels of R10 protein in WT, *te*, OE17 and OE65 seeds. (**g**) The phenotype of the *R10* RNAi line in the *te* background. Scale bar, 10 cm. (**h**) Western blot analysis shows that the level of R10 protein is reduced in the *te*^R10-RNAi^ plants compared with *te*. (**i**) Heights of 3-month-old WT, *te* and *te*^R10-RNAi^ plants. (**j**) The tiller number of 3-month-old WT, *te* and *te*^R10-RNAi^ plants. Values are means±s.d. in **i** and **j** (*n*=30 seedlings). ‘α-Tubulin' in **c** or ‘α-HSP82'in **f** and **h** indicate that roughly equal amounts of total plant extracts were used.

Previous studies have reported that Cdh1 (TE) mainly recognizes the destruction-box (D-box; RxxL) and KEN-box (Lys (K)-Glu (E)-Asn (N)) in substrate proteins to target them for ubiquitination and proteasome-mediated degradation^16^,^17^,^18^. To test whether TE may regulate ABA and GA signalling via targeted degradation of known ABA and GA signalling components, we searched for D-box and KEN-box sequences in key positive components of ABA signalling and negative components of GA signalling. Interestingly, we found a typical D-box region in the Lid loop one of both rice and *Arabidopsis* PYR/PYL/RCAR proteins (Supplementary Fig. 3). A cell-free degradation assay showed that all of the eight PYL/PYRs examined were effectively degraded by WT extracts, but their degradation was slowed down in the *te* mutant extracts. Strikingly, OsPYL/RCAR2, OsPYL/RCAR9 and OsPYL/RCAR10 were very stable in *te* plant extracts. Further, treatment with proteasome inhibitor MG132 effectively blocked their degradation (Supplementary Fig. 4). To verify whether these OsPYL/RCARs proteins are authentic substrates of APC/C^TE^, we selected OsPYL/RCAR10 (abbreviated as R10 hereafter) for further detailed analyses. R10 shares the highest homology to *Arabidopsis* PYL1 and PYR1 (ref. 5) and is widely expressed in all rice tissues (Supplementary Fig. 5a). Similar to the *Arabidopsis* RCAR1 (ref. 4) and PYR1 (ref. 5) proteins and rice TE protein^16^,^18^, the R10-GFP fusion protein was localized in both the nucleus and cytoplasm (Supplementary Fig. 5b). As expected, the *R10-GFP* overexpression lines displayed an ABA hypersensitive phenotype, while reducing *R10* expression via RNA interference (RNAi) caused a decreased sensitivity to ABA (Supplementary Fig. 5c–i). Thus, R10 likely functions as an authentic ABA receptor in rice, like its *Arabidopsis* counterpart^5^. Both bimolecular fluorescence complementation (BiFC) and Co-IP assays showed that TE physically interacted with R10 (Fig. 2a,b). In addition, we found that degradation of R10 or R10-GFP could be effectively blocked by two different proteasome inhibitors, MG132 or MG115 (Fig. 2c), whereas the mutant His-R10-m protein (with a mutated D-box; RLDL→ALDA) remained relatively stable in WT plant extracts compared with the WT His-R10 protein (Fig. 2d). Moreover, an *in vitro* ubiquitination assay showed that His-R10 was polyubiquitinated more efficiently by WT plant extracts than by *te* plant extracts (Fig. 2e). Further, a western blot with anti-R10 antibody showed that R10 over accumulated in *te* plants but was reduced in OE17 and OE65 plants, compared with WT plants (Fig. 2f). Finally, reducing *R10* expression via RNAi in *te* plants rescued the seedling height and seed germination defects but not the tillering phenotype of *te* mutant (Fig. 2g–j and Supplementary Fig. 6), indicating that *R10* acts downstream of *te* and is specifically involved in TE-mediated ABA responses. Together, these results support the notion that APC/C^TE^ represses ABA signalling by targeted proteasomal degradation of R10 (and possibly some other OsPYL/RCARs proteins as well).

### SnRK2 kinases inhibit the activity of APC/C^TE^

**Figure 3:**
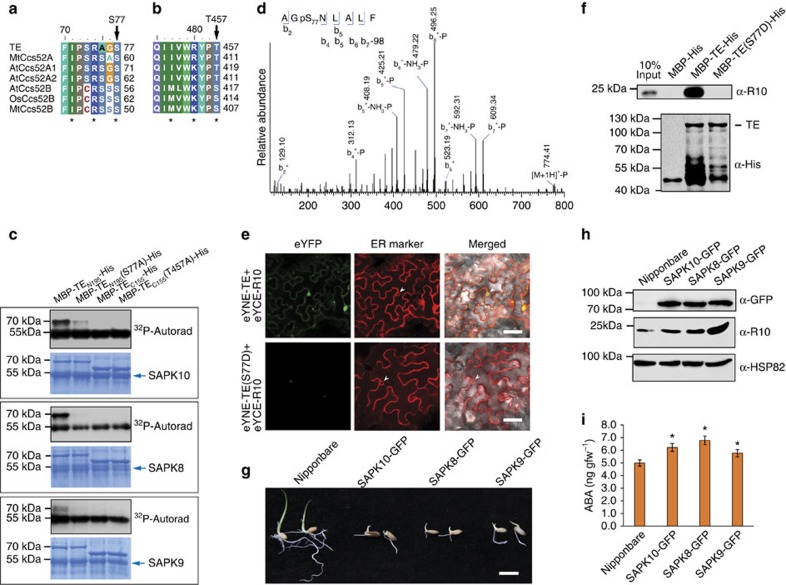
SnRK2s repress the activity of APC/C^TE^. (**a** and **b**) Analysis of the TE protein sequence identifies two conserved sites, S77 (**a**) and T457(**b**), that are potential sites for phosphorylation by SnRK2s. * shows the key amino acids needed for SnRK2 recognition. (**c**) *In vitro* phosphorylation assay shows that the TE N-terminus, MBP-TE_N195_, which contains the S77 site, but not the MBP-TE_N195_(S77A) mutant, is phosphorylated by all three rice SnRK2s (SAPK8, SAPK9 and SAPK10); whereas neither MBP-TE_C155_, which contains the T457 site, nor the mutant MBP-TE_C155_(T457A), are phosphorylated by any of the three SnRK2s. (**d**) The phosphorylated TE pS77 peptide corresponding to residues 75–82. Observed b-ions and those resulting from the neutral loss of H_3_PO_4_ (−98 Da), or both H_3_PO_4_ and NH_3_ (−115 Da) are indicated. (**e**) BiFC analysis shows the interactions between TE or TE (S77D) and R10. White arrowheads show the nuclear membrane. Scale bar, 100 μm. (**f**) *In vitro* pull-down assay shows that MBP-TE-His, but not the mutant MBP-TE(S77D)-His, pulls down R10 (upper panel) from *te* plant extracts. The lower panel shows that roughly equal amounts of MBP-His, MBP-TE-His and MBP-TE (S77D)-His proteins were used. (**g**) The *SAPK10-GFP*, *SAPK8-GFP* and *SAPK9-GFP* overexpression lines showed delayed germination and seedling growth compared to the Nipponbare vector control. Scale bar, 1 cm. (**h**) Western blot analysis shows the levels of R10 protein in Nipponbare (vector control), *SAPK10-GFP*, *SAPK8-GFP* and *SAPK9-GFP* overexpression rice lines. The ‘α-HSP82' signal shows that roughly equal amounts of total plant extract were used. (**i**) Measurement of ABA levels in Nipponbare (vector control), *SAPK10-GFP*, *SAPK8-GFP* and *SAPK9-GFP* overexpression rice lines. Values are means±s.d. (*n*=4 replicates). All plants are 10-day-old. Student's *t*-test analysis indicated a significant difference (compared with Nipponbare, *P<0.05). gfw, gram fresh weight.

An integral positive component of ABA signalling, the SnRK2 kinases, phosphorylate S/T residues in the RXXS/T domain of their substrates^19^. We found two conserved SnRK2s recognition sites (S77 and T457) in the TE protein (Fig. 3a,b). Notably, it has been shown that the kinase activities of three rice SnRK2s (SAPK10, SAPK8 and SAPK9) were induced by ABA^19^. BiFC assays showed that TE physically interacted with SAPK8, SAPK9 and SAPK10 and yeast two-hybrid assays showed that TE interacted with SAPK8 and SAPK10 (Supplementary Fig. 7). Further, an *in vitro* phosphorylation assay with MBP-fusion proteins showed that the WT N-terminal peptide of TE (TE_N195_, containing the S77 site) could be clearly phosphorylated by SAPK10, SAPK8 or SAPK9, but TE_N195_(S77A) in which S77 was mutated, was only slightly phosphorylated by SAPK10 (Fig. 3c). Further, LC-MS/MS analysis detected a phosphate group on S77 of MBP-TE_N195_ protein phosphorylated *in vitro* (Fig. 3d). These results suggest that TE is a substrate of SnRK2s and that S77 is a likely phosphorylation site.

To test whether TE phosphorylation affects its association with R10, we performed BiFC assay and *in vitro* pull-down. We found that MBP-TE, but not the phosphomimetic mutant MBP-TE(S77D), bound to R10 (Fig. 3e,f), suggesting that phosphorylating S77 of TE may block its interaction with R10. Consistent with this, the *SAPK8-GFP*, *SAPK9-GFP* and *SAPK10-GFP* overexpression lines all exhibited delayed germination and reduced seedling growth, and accumulated more R10 compared with WT plants (Fig. 3g,h). Intriguingly, we also found that *SAPK8-GFP*, *SAPK9-GFP* and *SAPK10-GFP* overexpression lines accumulated slightly higher levels of ABA compared with WT plants (Fig. 3i). On the basis of these results, we suggest that SnRK2s positively regulate ABA signalling by stabilizing the OsPYL/RCAR receptors via phosphorylation of TE on one hand, and upregulating ABA biosynthesis on the other hand.

### Opposite effects of ABA and GA on the degradation of R10

**Figure 4:**
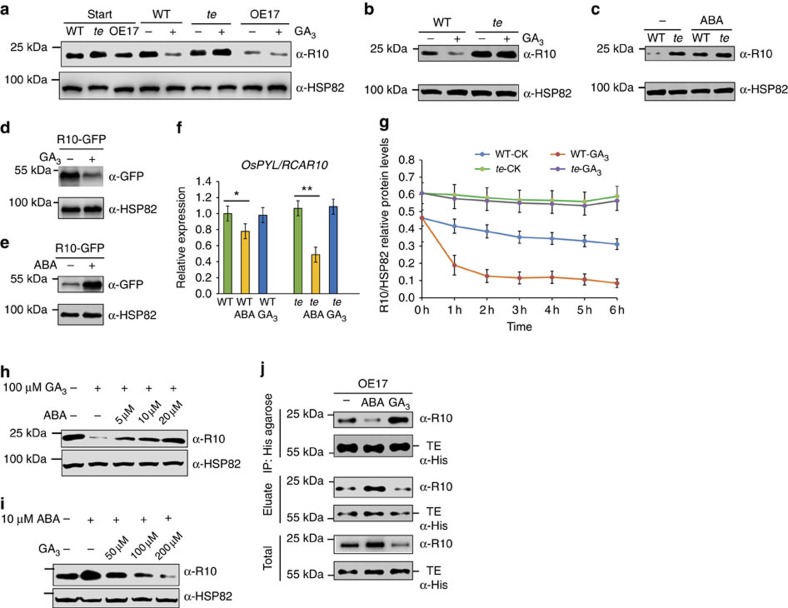
Opposite effects of GA and ABA on APC/C^TE^-mediated degradation of R10. (**a**) Seeds of WT, *te* and OE17 were imbibed for 24 h, then treated for 3 h with 100 μM GA3. Western blot analysis shows the corresponding levels of R10 protein in WT, *te* and OE17 seeds. (**b**) 100 μM GA_3_ treatment for 3 h induces degradation of R10 in WT, but not in *te* plants. (**c**) 10 μM ABA treatment for 3 h induces accumulation of R10 protein in WT plants. (**d**) Treatment for 3 h with 100 μM GA_3_ induces degradation of R10-GFP in the *R10-GFP* overexpression lines. (**e**) Treatment for 3 h with 10 μM ABA induces accumulation of R10-GFP protein in the *R10-GFP* overexpression lines. (**f**) A unit of 100 μM ABA but not GA_3_ treatment for 12 h reduced the expression level of *OsPYL/RCAR10* in both WT and *te* plants. Student's *t*-test analysis indicated a significant difference (*P<0.05, **P<0.01). (**g**) Degradation kinetics analysis of R10 protein. Seeds of WT and *te* were imbibed for 24 h, then treated with 100 μM GA3. The relative abundance of R10 over the time course was determined based on the R10/HSP82 ratios. (**h**) Induced degradation of R10 by 100 μM GA_3_ was repressed gradually by application of increased amounts of ABA in WT plants. (**i**) Induced accumulation of R10 by 10 μM ABA was reduced by application of increased amounts of GA_3_ in WT plants. (**j**) Co-IP assay showing the quantities of TE and R10 proteins pulled down from the extracts of 1-week-old OE17 plants treated with 10 μM ABA or 100 μM GA_3_ by equal amounts of His-agarose. ‘Eluate' shows the quantities of TE and R10 proteins that were not pulled down by His-agarose. ‘Total' shows the starting quantities of TE and R10 proteins that were used in the Co-IP assay. The ‘α-HSP82' signals in **a**–**e**, **h** and **i** show that roughly equal amounts of total plant extracts were used. Values are means±s.d. (*n*=3 replicates) in **f** and **g**. Note: The seeds or seedlings were pre-treated for 1 h with 1 mM cycloheximide before phytohormone treatment in **a**–**e** and **g**–**i**.

Next, to explore how APC/C^TE^ mediates the antagonism between ABA and GA, we compared the effect of GA treatment on R10 degradation in WT and *te* plants. All samples were pre-treated with 1 mM cycloheximide for 1 h to inhibit *de novo* protein biosynthesis and then treated with GA_3_ or ABA, respectively. Notably, GA_3_ effectively induced degradation of R10 in WT seeds and plants; but R10 remained stable in *te* seeds and plants (Fig. 4a,b and Supplementary Fig. 8a,b), and became less stable in OE17 seeds regardless of the treatments (Fig. 4a and Supplementary Fig. 8a). In contrast, ABA treatment stabilized R10 in WT plants (Fig. 4c). Similarly, GA_3_ induced the degradation of R10-GFP, while ABA stabilized R10-GFP proteins in the transgenic plants overexpressing *R10-GFP* (Fig. 4d,e). qRT–PCR analysis showed that after 12 h treatment, ABA reduced while GA_3_ did not substantially affect *R10* mRNA expression (Fig. 4f). A degradation kinetics assay further showed that GA_3_ markedly accelerated the degradation of R10 in WT seeds but not in *te* seeds (Fig. 4g). Moreover, we found that the induced degradation of R10 by GA_3_ could be gradually inhibited by application of increased amounts of ABA, and conversely, the induced accumulation of R10 by ABA could be reduced gradually by application of increased amounts of GA_3_ (Fig. 4h,i). These results suggest that ABA and GA_3_ act antagonistically to stabilize and destabilize the R10 protein, respectively, and APC/C^TE^ is required for GA-promoted degradation of R10. Consistent with this notion, we found that ABA inhibited while GA_3_ promoted the interaction between TE and R10 (Fig. 4j).

**Figure 5:**
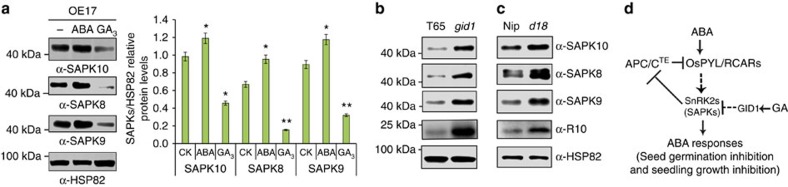
Antagonism of GA on ABA signalling by GA-promoted degradation of SnRK2s. (**a**) Treatment for 3 h with 100 μM GA_3_ reduces, while treatment with 10 μM ABA increases the levels of SAPK10, SAPK8 and SAPK9 proteins in OE17 plants. Left panel shows a representative western blot. Right panel shows the SAPKs/HSP82 relative protein level corresponding to left panel. Values are means±s.d. (*n*=3 replicates). Student's *t*-test analysis indicated a significant difference (compared with corresponding control (CK), *P<0.05, **P<0.01). Note: The seeds or seedlings were pre-treated for 1 h with 1 mM cycloheximide before phytohormone treatment. (**b** and **c**) Western blot analysis showing the levels of SAPK10, SAPK8, SAPK9 and R10 proteins in WT Taichung 65 (T65) and its *GA-insensitive dwarf1* mutant (*gid1*) (**b**) or in WT Nipponbare (Nip) and its GA biosynthesis mutant *dwarf 18* (*d18*) (**c**). (**d**) A model shows that the SnRK2-APC/C^TE^ regulatory module underlies the antagonism between GA and ABA. Dashed arrows or bars represent indirect action and solid arrows or bars represent direct action. The ‘α-HSP82' signals in **a**–**c** show that roughly equal amounts of total plant extracts were used.

To further investigate whether the antagonistic action of GA and ABA on the interaction between TE and R10 is mediated by SnRK2s, we analysed their effects on SnRK2 activity. We found that GA_3_ treatment reduced the accumulation of SAPK10, SAPK8 and SAPK9 proteins (Fig. 5a and Supplementary Fig. 9), while ABA treatment increased the accumulation of SAPK10, SAPK8 and SAPK9 proteins (Fig. 5a). Consistent with this, SnRK2 protein levels were also higher in the *GA-insensitive dwarf1* mutant (*gid1*)^13^ and GA biosynthesis mutant *dwarf 18* (*d18*)^20^,^21^, compared with WT plants (Fig. 5b,c). Further, treatment with the GA biosynthesis inhibitor Paclobutrazol also caused over-accumulation of SnRK2 proteins (Supplementary Fig. 9). In addition to stabilizing SAPK10, SAPK8 and SAPK9 proteins, ABA treatment also promoted the expression of *SAPK10* and *SAPK8* mRNA (Supplementary Fig. 10). On the basis of these results, we propose that GA promotes the interaction between TE and R10, and subsequent degradation of R10 by reducing SnRK2 activity while ABA inhibits their interaction by increasing SnRK2 activity at both transcriptional and post-transcriptional levels.

## Discussion

Previous studies have shown that DELLA proteins represent a regulatory hub that mediates the repression of ABA on GA signalling in plants^15^,^22^,^23^,^24^,^25^,^26^. On the basis of the results presented in this study, we suggest that the SnRK2s-APC/C^TE^ regulatory module represents a new signalling hub mediating the antagonistic action of GA on ABA signalling in higher plants. We propose that perception of ABA by the OsPYL/RCAR receptors activates SnRK2s, leading to phosphorylation of TE and disruption of the interaction between TE and ABA receptors, thus stabilizing the OsPYL/RCAR receptors and further enhancing ABA responses through a positive feedback mechanism (Fig. 5d). Although ABA can repress the expression of *R10* mRNA by a negative feedback mechanism (Fig. 4f), it does not destabilize R10 and SnRK2 proteins. Instead, R10 is destabilized by GA in the WT plants or by overexpression of *TE* in OE17 seeds. Further, R10 protein is stable in *te* plants, GA signalling and biosynthetic mutants (Figs 4a–c and 5b,c). Together, these results suggest that ABA mainly positively regulates ABA signalling through a post-transcriptional regulatory mechanism. On the other hand, we suggest that GA can reduce ABA signalling by promoting the interaction between TE and OsPYL/RCARs by reducing SnRK2 activity, and causing subsequent proteasomal degradation of OsPYL/RCARs (Fig. 5d). The self-enhancing effect of ABA on its biosynthesis^23^ and signalling and desensitization by GA is in sharp contrast with other known signalling pathways in both plants and animals reported so far^27^,^28^,^29^,^30^,^31^,^32^,^33^,^34^. In most reported cases, organisms have adopted a self-repression mechanism to attenuate many signalling pathways after they are activated by corresponding signal molecules, and long-term activation of these signalling pathways will have catastrophic consequences^29^,^30^,^31^,^35^. Unlike mobile animals, sessile plants cannot evade but have to tolerate unfavourable environmental conditions such as drought, salinity or cold. Thus, it is conceivable that the self-enhancing mechanism of ABA responses might offer an advantageous tactic for plants to survive in long-term stressful conditions. In line with this proposition, it was recently reported that proteasome-mediated degradation of the ABA receptor PYL8 is also counteracted by ABA in *Arabidopsis*^36^, suggesting conservation of such a regulatory mechanism in higher plants. When the environmental conditions became more favourable, the GA pathway may be activated to promote the degradation of ABA core signalling components (ABA receptors and SnRK2s) and DELLA proteins^13^, which would allow plants to resume normal growth and development. Antagonistic action of GA and ABA thus may serve as a ‘rheostat' to fine tune plant growth and development in response to the fluctuating environments.

It is worth noting that among the eight rice ABA receptors examined in this work, OsPYL/RCAR2, OsPYL/RCAR9 and R10 are very stable in *te* plant extracts (Supplementary Fig. 4), suggesting that APC/C^TE^ is the major E3 ligase for their degradation; but, other five OsPYL/RCARs were still reduced in *te* plant extracts compared with ‘Input' (Supplementary Fig. 4), suggesting that besides APC/C^TE^, other E3 ligases are likely involved in the degradation of these five ABA receptors. Consistent with this proposition, recent studies reported that in *Arabidopsis*, the RPN10 subunit of 26S proteasome, the substrate adaptor DDA1 of a multi-subunit E3 ligase and a single subunit E3 ligase RSL1 all target specific PYL proteins for proteasomal degradation^36^,^37^,^38^,^39^. The employment of multiple E3 ligases for proteasomal degradation of the ABA receptor proteins possibly enables the plants to more effectively respond to different developmental or external signals and adds additional complexity of ABA signalling regulation in higher plants. Identification and functional studies of other unknown E3 ligases will lead to a better understanding of ABA signalling mechanism and its crosstalk with other signalling pathways.

## Methods

### Plant materials and growth conditions

The WT, *te* mutant and *TE* overexpression transgenic lines OE17 and OE65 used in this study were described previously^16^. Except when indicated otherwise, rice plants were cultivated in an experimental field at Beijing in the natural growing seasons. For qRT–PCR assays, phytohormone treatments, ABA analyses and Co-IP assays, the seedlings of WT, *te*, OE17 and OE65, were grown in climate chambers (HP1500GS, Ruihua) at 70% humidity, under long-day conditions with a photocycle of 14.5 h light (30 °C) and 9.5 h darkness (25 °C). Light was provided by fluorescent white-light tubes (400–700 nm, 250 μmol m^−2^ s^−1^).

### Germination and seedling growth assay

For the seed germination assay, dehulled rice (Oryza sativa) seeds from WT, various mutant and transgenic rice lines were first surface sterilized in 70% ethanol for 1 min and washed once with sterilized water. Then, seeds were immersed in NaClO for 40 min, and subsequently were washed at least five times with sterilized water. Rinsed seeds were planted on half-strength MS medium (pH 6.0) (M524, Phyto Technology Lab) supplemented with 0.4% Gelzan (G3251, Sino Industrial) and various concentrations of (±) ABA or GA_3_. The seeds were then placed in a growth chamber (Ruihua) with a 14.5/9.5-h light/dark cycle at 30/25 °C. Germination was considered complete when the coleoptile was 5 mm long. Every experiment was repeated three times, with 30 seeds per sample.

For drought stress, WT and *te* mutant plants were grown in small pots with the same amount of soil. After growth for 3 weeks, drought was imposed by withdrawing irrigation for 10 days. On the eleventh day after withdrawing irrigation, surviving seedlings were photographed and counted.

### Vector construction and plant transformation

To generate the DNA constructs, *pUBI*::*OsPYL/RCAR10-GFP*, *pUBI*::*SAPK10-GFP*, *pUBI*::*SAPK8-GFP* and *pUBI*::*SAPK9-GFP*, we amplified full-length coding sequence (CDS) of *OsPYL/RCAR10*, *SAPK10, SAPK8* and *SAPK9* with the primers shown in Supplementary Table 1. The PCR products were then cloned into the binary vector, pCUBI1390, which has a GFP insertion, with the In-Fusion Advantage PCR Cloning Kit (Cat: PT4065, Clontech). To generate the *OsPYL/RCAR10* RNAi construct, the *OsPYL/RCAR10* CDS was amplified with the primers shown in Supplementary Table 1, and the PCR product was inserted into the LH-FAD2-1390 RNAi vector. The resultant constructs were introduced into the rice Nipponbare variety or *te* mutant by *Agrobacterium tumefaciens*-mediated transformation.

### Antibody preparation and western blot analysis

The complete CDSs of OsPYL/RCAR10, SAPK10, the CDS of 217–361aa of SAPK8, or the CDS of 207–371aa of SAPK9 were amplified, respectively, with the primers shown in Supplementary Table 2, and the PCR products were inserted into the pGEX-4T-1 vector to express the GST-OsPYL/RCAR10, GST-SAPK10, GST-SAPK8 or SAPK9 fusion proteins, respectively. Antibodies against OsPYL/RCAR10, SAPK10, SAPK8 or SAPK9 were prepared by immunizing rabbits with the GST-OsPYL/RCAR10, GST-SAPK10, GST-SAPK8 or GST-SAPK9 fusion proteins, respectively, and then affinity purified with corresponding GST-OsPYL/RCAR10, GST-SAPK10, GST-SAPK8 or GAT-SAPK9 fusion proteins, respectively. Western blots were performed with the purified antibodies at 1:2,000 dilution and visualized with enhanced chemiluminescence reagent (GE Healthcare). The antibody against OsCDC27 (at 1:2,000 dilution) was described previously^16^. The antibodies of anti-His (Cat: D291-7, at 1:2,000 dilution), anti-GFP (Cat: 598-7, at 1:2,000 dilution), anti-Flag (Cat: M185-7, at 1:5,000 dilution) and anti-α-tubulin (Cat: PM054-7, at 1:1,000 dilution) were purchased from Medical & Biological Laboratories CO..Ltd. (MBL) and the antibodies against HSP82 (Cat: AbM51099-31-PU, at 1:10,000 dilution) were purchased from Beijing Protein Innovation. The uncropped full scans of all western blot/gel images are presented in Supplementary Figs 11 and 12.

All western blot experiments were repeated at least three times, essentially with the same conclusions, and representative results are shown. Quantification of western blots was conducted according to Saijo *et al*.^40^. Briefly, band intensities of R10, input (start quantity) and HSP82 (loading control for total lysates), were measured with ImageJ (http://rsb.info.nih.gov/ij/). Relative band intensities were then calculated using the ratio of R10/input or R10/HSP82 for each western blot panel.

### Construction of protein expression plasmids

The CDS encoding the N-terminal 195aa (*TE*_*N195*_) and the C-terminal 155 aa (*TE*_*C155*_) of TE, as well as the mutant forms, *TE-N195*(*S77A*), *TE-C155* (*T457A*), *TE*(*S77A*) and *TE*(*S77D*) were amplified using the primers shown in Supplementary Table 2. The PCR products were cloned into the pMAL-C2x vector (NEB) with the In-Fusion Advantage PCR Cloning Kit (Cat: PT4065, Clontech) to generate the following plasmids: pMAL-C2x::MBP-TE-N195-His, pMAL-C2x::MBP-TE-C155-His, pMAL-C2x::MBP-TE-N195 (S77A)-His, pMAL-C2x::MBP-TE-C155 (T457A)-His, pMAL-C2x::MBP-TE-His, pMAL-C2x::MBP-TE (S77A)-His and pMAL-C2x::MBP-TE (S77D)-His. The CDS for *OsPYL/RCAR1* to *OsPYL/RCAR10* or the mutant, *OsPYL/RCAR10-m* (with a D-box mutation), were amplified with the primers shown in Supplementary Table 2. The PCR products were inserted into the pET28a vector to express the His-OsPYL/RCAR1 through His-OsPYL/RCAR10 proteins in *Escherichia coli* (BL21).

### *In vitro* pull-down assays

For the *in vitro* pull-down assay of R10 for Fig. 3f, total proteins were extracted from 1-week-old *te* seedlings in a degradation buffer^41^. Then, roughly equal amounts of purified MBP-His, MBP-TE-His and MBP-TE(S77D)-His fusion proteins (about 1 μg) were affixed to Amylose Resin (NEB), and incubated in 400 μl *te* seedling extracts (containing 1.6 mg total proteins) for each assay, with 50 μM MG132, a proteasome inhibitor. The mixture was gently shaken at 28 °C for 30 min. The pull-down assay was performed as reported previously^42^,^43^, with anti-His or anti-R10 antibodies at 1:2,000 dilution.

### Cell-free degradation assays

Total protein extracts were prepared from *te*, WT, Nipponbare and OsPYL/RCAR10-GFP transgenic seedlings, as described previously^16^. Then, MG132, MG115, PMSF, LEU or APR were selectively added to various *in vitro* degradation assays, as indicated. For the degradation assays, equal amounts (about 500 ng) of the purified His-OsPYL/RCAR1 through His-OsPYL/RCAR10 proteins and the His-OsPYL/RCAR10-m protein were each incubated in 50 μl of rice total protein extract (containing about 200 μg total proteins). The mixtures were incubated at 28 °C for 1 h, and the samples were processed for western blot analysis to determine the abundances of OsPYL/RCAR10, OsPYL/RCAR10-GFP, His-OsPYL/RCAR1 to His-OsPYL/RCAR10 and His-OsPYL/RCAR10-m; membranes were probed with anti-OsPYL/RCAR10, anti-GFP or anti-His antibodies at 1:2,000 dilution.

### Yeast two-hybrid assays

The CDS of *TE* was cloned into the Y2H ‘prey' vector, pGADT7 (Clontech). The CDS of *SAPK10*, *SAPK8* and *SAPK9* were cloned into the Y2H ‘bait' vector, pGBKT7 (Clontech). Bait and prey constructs were co-transformed into yeast (*Saccharomyces cerevisae*) strain, AH109. The yeast two-hybrid assay was performed according to the manufacturer's instructions.

### *In vitro* ubiquitination assays

Purified His-OsPYL/RCAR10 proteins, bound to Ni-NTA-Agarose (Novagen), were incubated at 28 °C with equal amounts of crude rice seedling extracts in a buffer containing 25 mM Tris-HCl pH 7.5, 10 mM MgCl_2_, 5 mM dithiothreitol (DTT), 10 mM NaCl, 10 mM ATP and 40 μM MG132. After incubating for the indicated intervals, the His-OsPYL/RCAR10 and the polyubiquitinated (Ubn) His-OsPYL/RCAR10-(Ubn) fusion proteins were added to SDS-PAGE loading buffer, then loaded onto an SDS-PAGE gel. Western blots were performed with antibodies against the polyubiquitin tail or His. The up-shifted bands of His-OsPYL/RCAR10 in Fig. 2e were confirmed to be the polyubiquitinated His-OsPYL/RCAR10, detected with anti-polyubiquitin antibodies (Cell Signaling, Catalogue No. 3936S) at 1:1,000 dilution.

### *In vitro* phosphorylation assays

The full-length CDSs of SAPK8, SAPK9 and SAPK10 were ligated into the pET28a vector; the CDS of the truncated TE was ligated into the pMAL-c2x vector (primers shown in Supplementary Table 2); the vectors were created with the In-Fusion Advantage PCR Cloning Kit (Takara). The proteins were expressed in *E. coli* and purified according to the user's manual. Phosphorylation assays were performed with 1 μg of recombinant fusion protein, MBP-TE_N195_-His, MBP-TE_N195_ (S77A)-His, MBP-TE_C155_-His or MBP-TE_C155_ (T457A)-His and 2 μg of His-SAPK protein in 30 μl of kinase buffer (40 mM Hepes, pH 7.5, 20 mM MgCl_2_, 2 mM DTT, 10 μCi [^32^P] γATP, 1 × proteinase inhibitor cocktail and 1 × phosphatase inhibitor cocktail), incubated at 30 °C for 1.5 h. The reaction was terminated by adding 6 μl sample buffer and heating at 100 °C for 5 min. After separation on a 10% SDS-PAGE gel, the gel was stained with Coomassie blue and imaged with a BIO-RAD Gel Doc XR+ imaging system. Then, the gel was exposed to GE Amersham hyperfilm MP film for detecting phosphorylated proteins.

### BiFC assays

The TE-eYNE plasmid was reported previously^16^. The CDS of *SAPK10*, *SAPK8* and *SAPK9* were amplified with primers listed in Supplementary Table 3 and cloned into the vector pSPYCE(M) (eYCE). For transient expression, *A. tumefaciens* strains (EHA105) carrying the BiFC constructs were used together with the p19 strain and ER marker, mCherry ER-rk CD3-959 (ref. 44), for infiltration of 5-week-old *Nicotiana benthamiana* leaves as described in Waadt and Kudla^45^. Infiltrated leaves were observed 48–72 h after infiltration using a laser confocal scanning microscope (ZEISS Microsystems LSM 700). The eYFP and mCherry fluorescent signals from the expressed fusion constructs were monitored sequentially. The excitation and detection wavelengths for eYFP and mCherry were 514 and 587 nm for excitation and 527 and 610 nm for detection, respectively.

### UPLC-MS/MS Analysis of ABA

Frozen rice samples were ground in liquid nitrogen with mortar and pestle. The internal standards 45 pmol ^2^H_6_-ABA (OlChemIm (Olomouc, Czech Republic)) were added to 100 mg of ground powder. The powder was extracted with 2 ml methanol and kept overnight at –20 °C, then centrifuged at 4 °C for 15 min at 18,000 r.p.m. The supernatant was collected, dried under nitrogen, then dissolved in 1 ml ammonia solution (5%). The crude extracts were further purified by Oasis MAX solid phase extraction (SPE) column (Waters (Milford, MA)), which had been sequentially preconditioned with 4 ml methanol, 4 ml water and 4 ml ammonia solution (5%). After the samples were loaded, SPE columns were sequentially washed with 4 ml ammonia solution (5%), 4 ml water and 4 ml methanol. ABA were eluted with 4 ml methanol contain 10 or 5% formic acid. The eluent was dried under nitrogen gas and finally dissolved in 200 μl water/methanol (20:80, v/v) for further UPLC/MS-MS analysis.

For UPLC/MS-MS analysis, LC system of Waters ACQUITY UPLC (Waters, Milford, MA) was used with a Waters ACQUITY UPLC BEH C18 column (2.1 × 100 mm inner diameter, 1.7 μm). ABA was separated with a mobile phase consisting of acetonitrile and water, both of which contained 0.05% acetic acid (v/v). The gradient run was at a flow rate of 0.5 ml min^−1^ with initial 15% acetonitrile, which was then increased to 40% in 5 min and further increased to 80% in the next 0.5 min. The injection volume for all samples was 5 μl and the column temperature was 35 °C. The UPLC system was coupled online with Waters Quattro Premier XE mass spectrometer (Micromass, Manchester, UK) equipped with an electrospray ionization source. The eletrospray capillary voltage was operated at 2.80 kV in the negative ion mode. The optimized parameters with electrospray ionization source obtained by infusion of the standard solution of 10 pmol μl^−1^ in acetonitrile/water (50:50, v/v) at 10 μl min^−1^ were the following: source temperature, 110 °C; desolvation temperature, 350 °C; desolvation gas flow, 600 l h^−1^; cone gas flow, 60 l h^−1^; multiplier, 650 V. Quantitative analysis was performed in multiple reaction monitoring (MRM) mode with four timesegmented scannings as reported previously^46^.

### qRT–PCR analyses

RNA was extracted from frozen samples with the RNAprep Pure Plant Kit (Tiangen) according to the manufacturer's instructions. qRT–PCRs were performed with the SYBR Premix Ex Taq RT–PCR kit (Takara), according to the manufacturer's instructions, with the primers listed in Supplementary Table 4.

### Phytohormone treatment analyses

One-week-old seedlings of WT, *te*, Nipponbare and *OsPYL/RCAR10-GFP*, *SAPK10-GFP*, *SAPK8-GFP*, *SAPK9-GFP* transgenic lines were grown in a growth chamber (Ruihua) under a 14.5/9.5-h light/dark cycle at 30 °C. These plants were subjected to ABA and GA_3_ treatment to determine their effects on the stability of OsPYL/RCAR10 and SnRK2 proteins. For phytohormone treatments, seedlings were placed in 50 ml tubes containing 5 ml of half-strength MS liquid medium supplemented with 1 mM cycloheximide (Inalco), 5 μl ethanol (control), 0.5 μl 100 mM ABA or 5 μl 100 mM GA_3_. Subsequently, the tubes were placed in a growth chamber (Ruihua) under a 14.5/9.5-h light/dark cycle at 30 °C for the indicated times. After phytohormone treatment, the seedlings were frozen in liquid nitrogen for further analysis. Western blots were performed with antibodies against OsPYL/RCAR10, SAPK10, SAPK8, SAPK9, GFP or HSP82, and signals were visualized with enhanced chemiluminescence reagent (GE Healthcare).

### Co-immunoprecipitation assays

For Fig. 2b, no phytohormone treatment was done. For Fig. 4j, 1-week-old OE17 plants were washed several times with water. Then, they were placed in 50 ml tubes containing 5 ml of half-strength MS liquid medium supplemented with either 5 μl ethanol, 0.5 μl 100 mM ABA or 5 μl 100 mM GA_3_. Subsequently, the tubes were placed in a growth chamber (Ruihua) under a 14.5/9.5-h light/dark cycle at 30 °C for 3 h. After phytohormone treatment, the seedlings were frozen in liquid nitrogen for further analysis.

For Co-IP assays, total proteins were extracted from 1-week-old *te* and OE17 seedlings in a RIPA buffer containing 50 mM Tris-HCl (PH 7.5), 150 mM NaCl, 10 mM MgCl_2_, 1 mM EDTA, 10% Glycerol, 50 μM MG132, 1x complete proteinase inhibitors (Roche), 1x PhosSTOP phosphatase inhibitors (Roche), 2 μM Staurosporine (Cell Signaling). Then, 200 μl His·Bind resin (Novagen) were incubated with 800 μl *te* or OE17 extracts (containing 3 mg total proteins) for each assay. The mixture was gently shaken at 4 °C for 15 min and then loaded on the His·Bind Columns (Novagen). The first eluate was collected for western blot analysis. Then the columns were washed two times with 1 ml RIPA buffer. The total protein extracts, first eluate and final resins were resolved in 1xSDS-PAGE sample buffer and western blots were conducted with anti-His or anti-OsPYL/RCAR10 antibodies at 1:2,000 dilution.

### LC-MS/MS analysis of phosphorylated TE peptides

The bands of *in vitro* phosphorylated MBP-TE_N195_ protein were excised from the PAGE gels, reduced with 25 mM DTT, and alkylated with 55 mM iodoacetamide. In-gel digestion was performed with trypsin (Promega) at 37 °C overnight, then sample was heated at 60 °C for 1 h to inactivate trypsin and further digested with Asp-N (Promega) at 37 °C overnight. The peptides were extracted twice with 0.1% (v/v) trifluoroacetic acid in 50% (v/v) acetonitrile aqueous solution for 30 min. The extracts were then centrifuged in a speed-vac to reduce the volume. After that, they were resolved in 0.1% trifluoroacetic acid water solution for Liquid Chromatography-Mass Spectrometry/Mass Spectrometry (LC-MS/MS) analysis.

For LC-MS/MS analysis, the digested peptides were separated by a 60-min gradient elution at a flow rate of 0.3 μl min^−1^ with the Dionex Ultimate 3000 Nano HPLC system that was directly interfaced with the Thermo Q Exective mass spectrometer. The analytical column was performed with a Thermofisher Acclaim Pepmap RSLC C18 column (150 mm × 75 μm × 2 μm). Mobile phase A consisted of 0.1% formic acid, and mobile phase B consisted of 80% acetonitrile and 0.08% formic acid. The Q Exactive mass spectrometer was operated in the data-dependent acquisition mode using Xcalibur2.1.3 software and there is a single full-scan mass spectrum in the Orbitrap (300–1,800 m/z, 17,500 resolution) followed by four targeted tandem mass spectrometry scans at 27% normalized collision energy. The mass of peptides with or without phosphorylation (M/Z 792.42 and 872.39, Z=1) was added into the inclusion list. The tandem mass spectrometry spectra from each LC-MS/MS run were searched against the selected database using Proteome Discovery searching algorithm (version 1.4).

## Additional information

**How to cite this article:** Lin, Q. *et al*. The SnRK2-APC/C^TE^ regulatory module mediates the antagonistic action of gibberellic acid and abscisic acid pathways. *Nat. Commun.* 6:7981 doi: 10.1038/ncomms8981 (2015).

## Supplementary Material

Supplementary Information Supplementary Figures 1-12 and Supplementary Tables 1-4
