# Supplementary material for: The SnRK2-APC/CTE regulatory module mediates the antagonistic action of gibberellic acid and abscisic acid pathways
Source: Nat Commun. 2015 Aug 14;6:7981. doi: 10.1038/ncomms8981 (PMC4557272; doi:10.1038/ncomms8981)
Supplement: Supplementary Information — Supplementary Figures 1-12 and Supplementary Tables 1-4 [file ncomms8981-s1.pdf]

Supporting Information

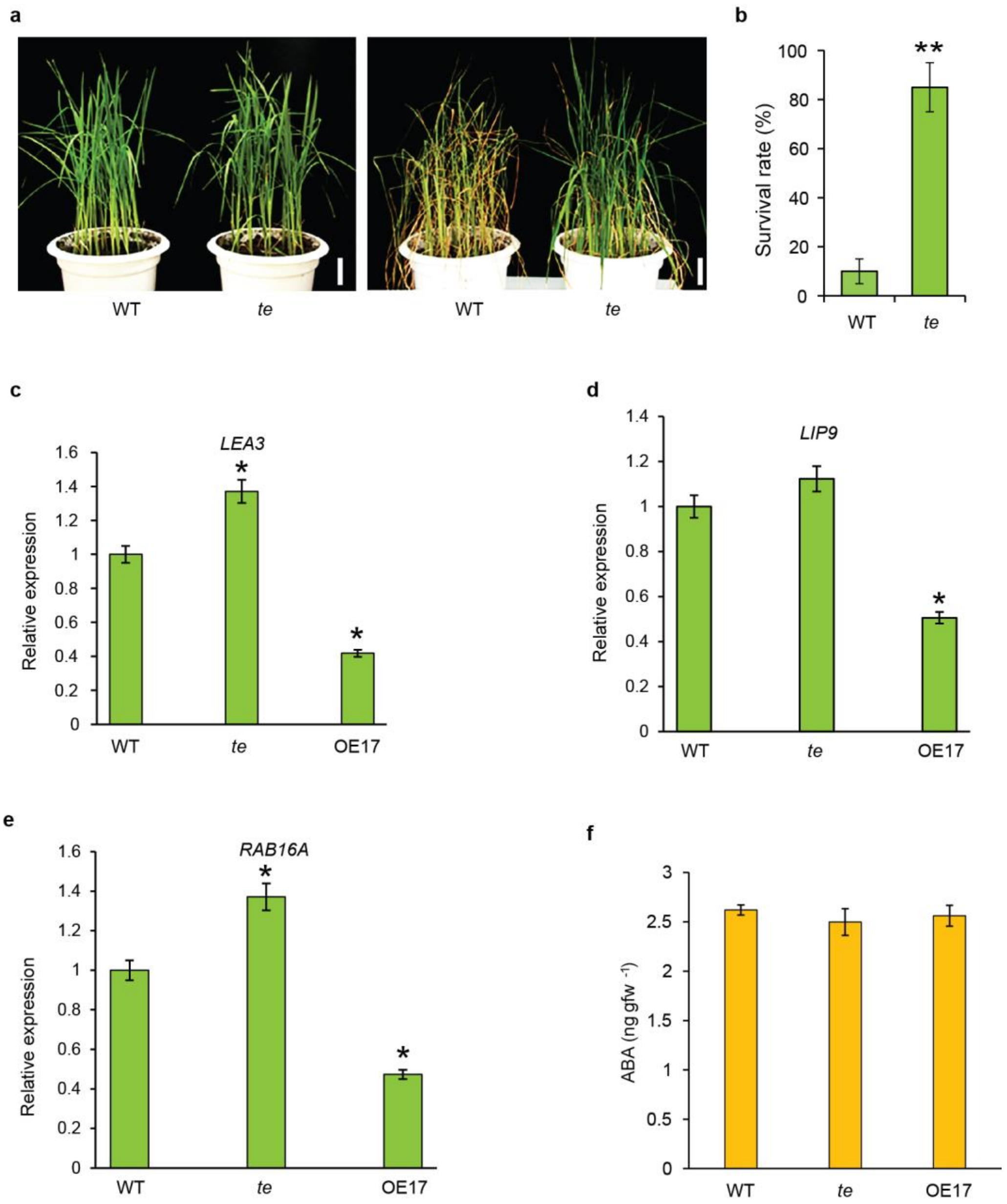

Supplementary Figure 1

The *te* mutant shows enhanced ABA responses.

(**a**) Enhanced tolerance to drought of *te* plants. Water was withheld from 3-week-old plants for 10 days, then the photograph was taken. The left and right panel show the seedlings before and after dehydration, respectively. Scale bars, 5 cm. (**b**) Increased survival rates of *te* plants under drought stress, compared to wild type plants (WT). Water was withheld for 10 days from 3-week-old plants and then the survivorship was counted. Values are means ± s.d. (n=30 seedlings). (**c-e**) The expression of *LEA3* (**c**), *LIP9* (**d**) and *RAB16A* (**e**) in WT, *te* and OE17 plants. (**f**) Measurement of ABA levels in WT, *te* and OE17 plants. Values are means ± s.d. (n=3 replicates) in **b-f**. All plants in **c-f** are one-week-old. Student's *t*-test analysis indicated a significant difference (compared to WT, $^*P<0.05$, $^{**}P<0.01$) in **b-e**. gfw, gram fresh weight.

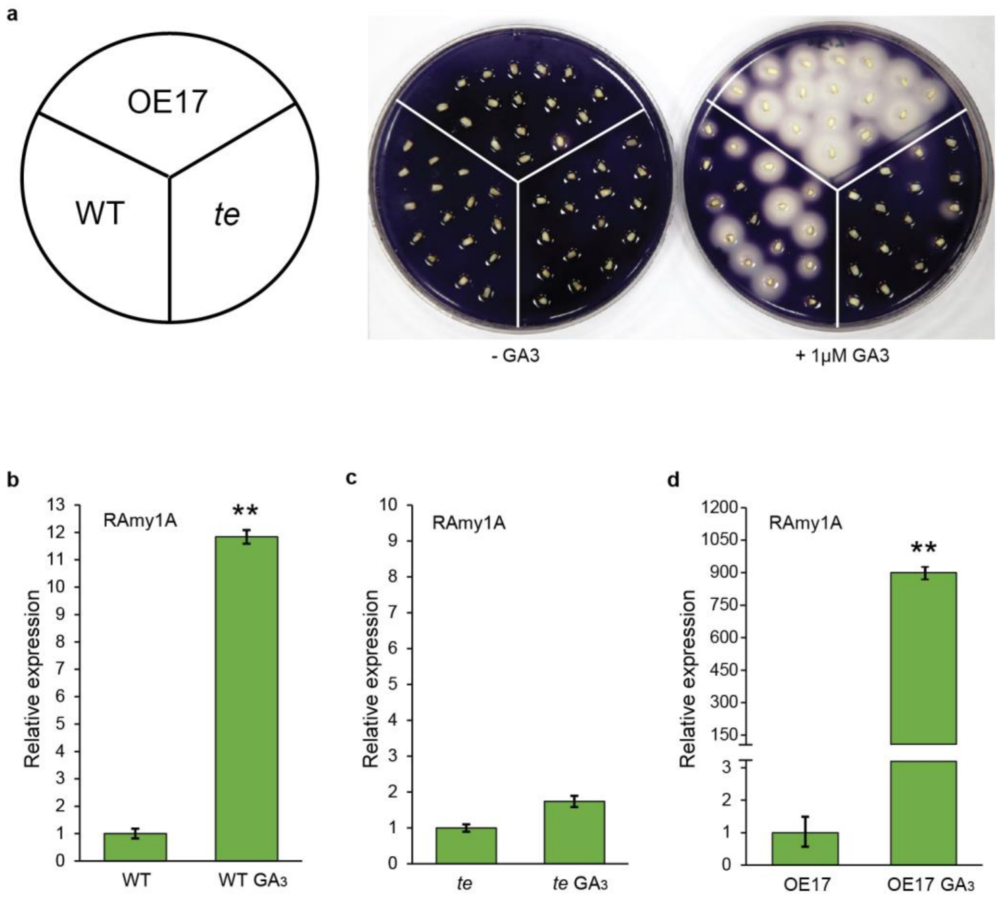

Supplementary Figure 2

The *te* mutant exhibits reduced sensitivity to GA.

(**a**) 1 μM GA_3 more effectively induces α-amylase secretion by OE17 and wild type (WT) than by *te* half seeds, as shown by the degradation of starches in the medium in these α-amylase assays. The half seeds were plated on medium for three days at 30 °C. (**b**-**d**) Treatment with 100 μM GA_3 for 12 hours effectively induces the expression of α-amylase gene *RAmy1A* in WT and OE17 plants but not in *te* plants. Values are means ± s.d. (n=3 replicates). Student's *t*-test analysis indicates a significant difference (compared with control, $^{**}P<0.01$).

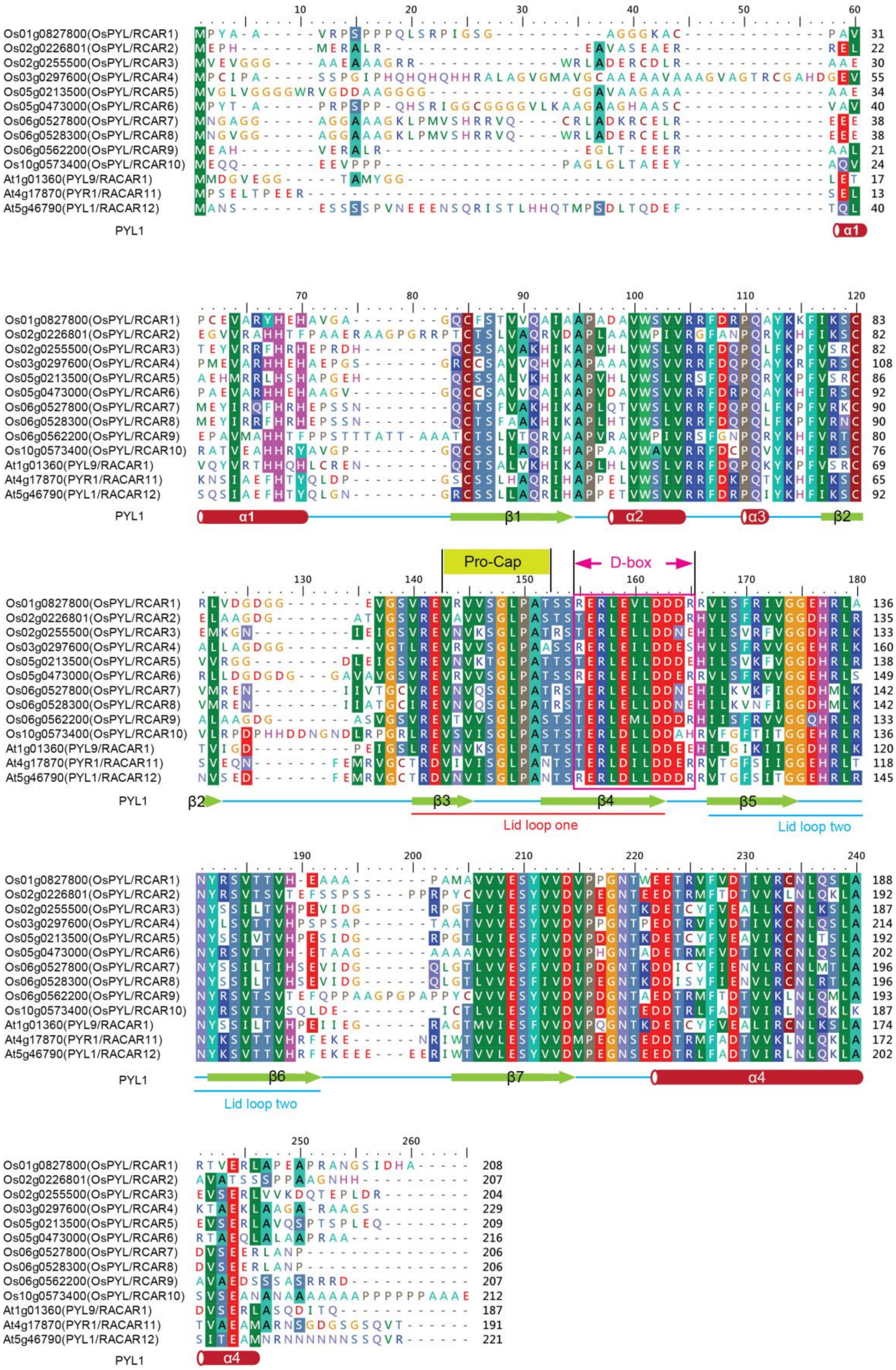

Supplementary Figure 3

Alignment of PYR/PYL/RCAR proteins.

Alignment of 10 rice PYR/PYL/RCAR proteins and 3 Arabidopsis PYR/PYL/RCAR proteins shows the conserved three-dimensional structural motifs. In addition, the conserved D-box mainly located in Lid loop one (composed of β3 and β4) is shown.

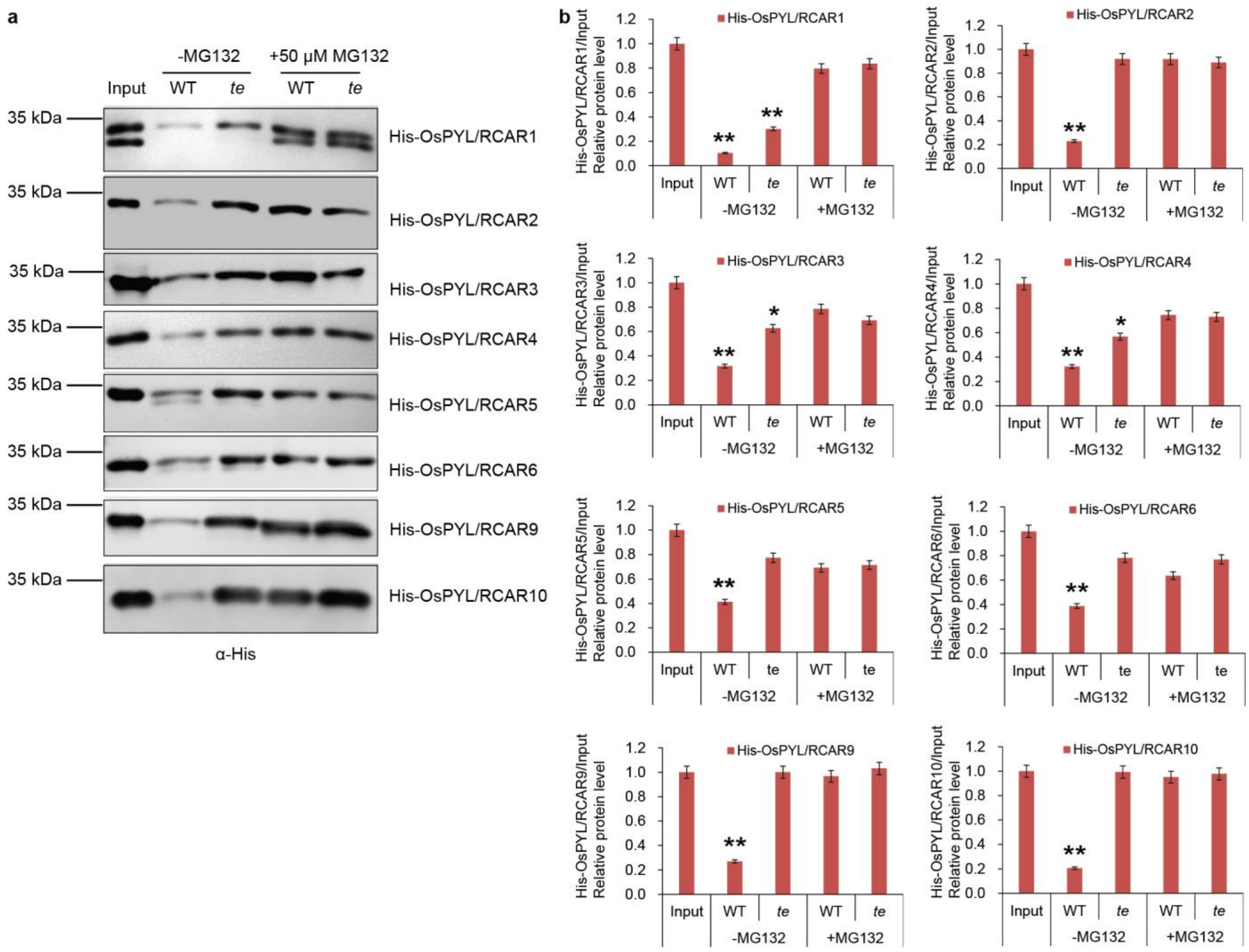

Supplementary Figure 4

Cell-free degradation assays of eight OsPYL/RCAR proteins.

(**a**)The eight His-OsPYL/RCAR proteins remained relatively stable after 1 hour in *te* plant extracts, compared to in wild type (WT) plant extracts. 50 μM MG132 effectively inhibited the degradations of eight His-OsPYL/RCAR proteins in WT plant extracts. 'Input' shows the amounts of each His-OsPYL/RCAR protein used in the assay. (**b**)The quantification analysis of relative His-OsPYL/RCARs/Input protein levels corresponding to Figure **a**. Values are means ± s.d. (n=3 replicates). Asterisks mark significant differences compared to Input according to Student's t test (*P < 0.05, **P < 0.01).

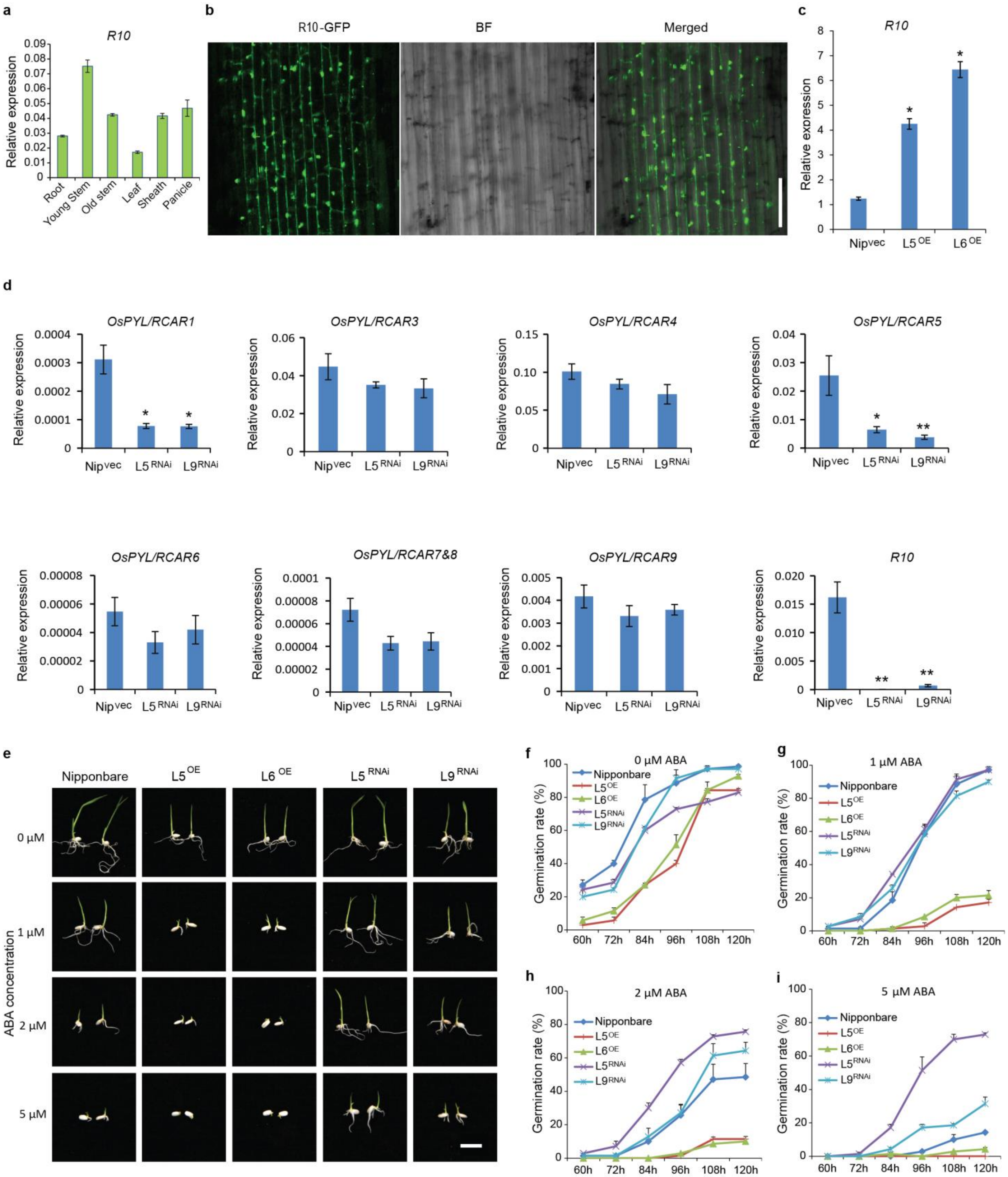

Supplementary Figure 5

Functional analysis of *R10*.

(**a**) qRT-PCR analysis shows that *R10* is expressed in many rice tissues, including roots, young stems, old stems, leaves, sheaths and panicles. (**b**) The R10-GFP fusion protein is localized in both the nucleus and cytoplasm of rice root cells. BF, bright-field image. (**c**) qRT-PCR analysis shows that the expression of *R10*

is upregulated in *R10-GFP* overexpression line L5^{OE} and L6^{OE} compared to Nipponbare (Nip) vector control. (**d**) qRT-PCR analysis shows the relative expression level of *OsPYL/RCAR1*, *OsPYL/RCAR3*, *OsPYL/RCAR4-10* in *R10* RNAi line L5^{RNAi} and L9^{RNAi} compared to Nipponbare (Nip) vector control. (**e**) ABA inhibition of growth of Nipponbare, L5^{OE}, L6^{OE}, L5^{RNAi} and L9^{RNAi} seedlings. Seeds of Nipponbare, L5^{OE}, L6^{OE}, L5^{RNAi} and L9^{RNAi} were grown on MS medium containing 0, 1, 2, or 5 μM ABA for 5 days. Photographs were taken on day 5. (**f-i**) Germination time courses on MS medium containing 0 μM ABA (**f**), 1 μM ABA (**g**), 2 μM ABA (**h**), or 5 μM ABA (**i**). Values are means ± s.d. (**a**, **c** and **d**, n=3 replicates; **f-i**, n=30 seedlings). Student's *t*-test analysis indicates a significant difference (compared with control, *P<0.05, ** P<0.01). Scale bars, 100 μm (**b**) and 1 cm (**e**).

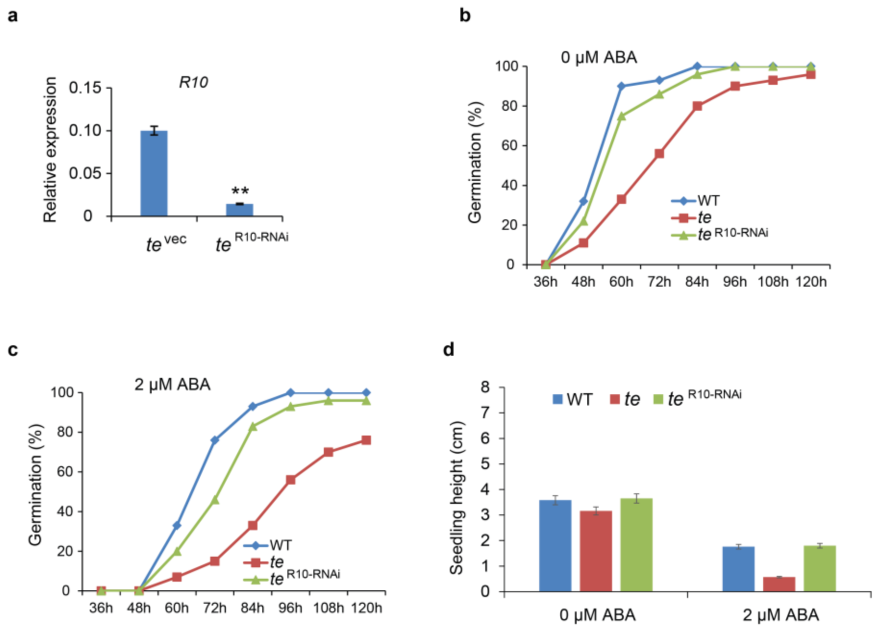

Supplementary Figure 6

Characterization of the *R10* RNAi line in the *te* background.

(**a**) qRT-PCR analysis shows the relative expression level of the *R10* in *R10* RNAi line in the *te* background ($te^{R10\text{-}RNAi}$) compared to the *te* vector control. Values are means ± s.d. (n=3 replicates). Student's *t*-test analysis indicates a significant difference (compared with control, ** P<0.01). (**b** and **c**) Germination time courses of WT, *te* and $te^{R10\text{-}RNAi}$ on MS medium containing 0 μM ABA (**b**) or 2 μM ABA (**c**). (**d**) Seedling heights of WT, *te* and $te^{R10\text{-}RNAi}$ grown for 5 days on MS medium containing 0 μM ABA or 2 μM ABA. Values are means ± s.d. in **b-d** (n=30 seedlings).

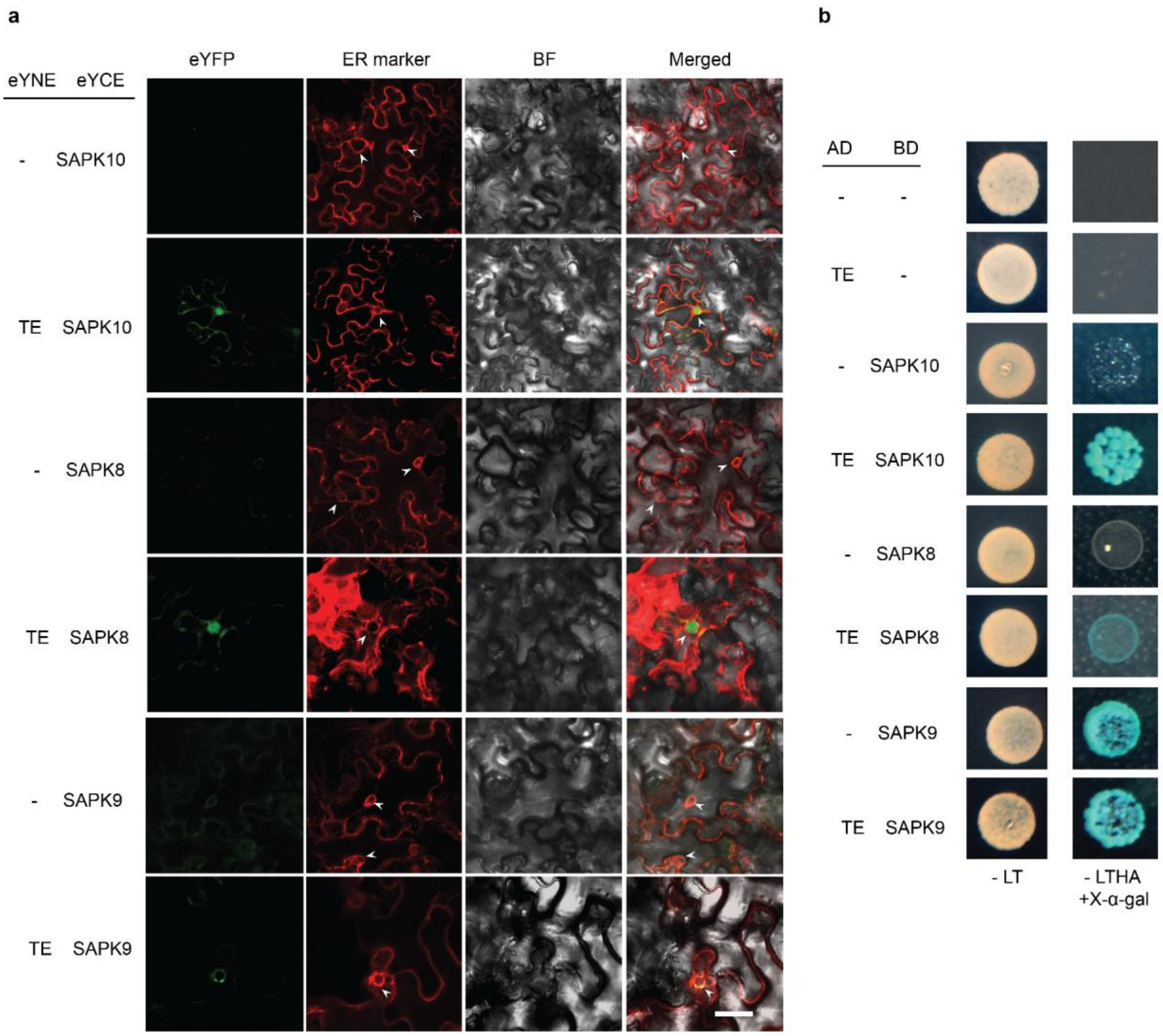

Supplementary Figure 7

TE interacts with three rice SnRK2 proteins (SAPK10, SAPK8 and SAPK9) *in vivo*.

(**a**) BiFC analysis showing interactions between TE and SAPK10, SAPK8 or SAPK9 in *N. benthamiana* leaf epidermis cells. eYFP, eYFP fluorescence; ER marker, a fluorescent marker protein localized in endoplasmic reticulum; BF, bright-field image. White arrowheads indicate the nuclear membrane. Scale bar, 50 μm. (**b**) Yeast two-hybrid assay shows that TE interacts with SAPK10 or SAPK8 but BD-SAPK9 shows strong auto-activation. Yeast transformants were plated on the control medium (SD-Leu/-Trp (-LT)) and selective medium (SD-Leu/-Trp/-His/-Ade plus X-α-gal) (-LTHA+X-α-gal). AD, activating domain; BD, binding domain; SD, synthetic dropout.

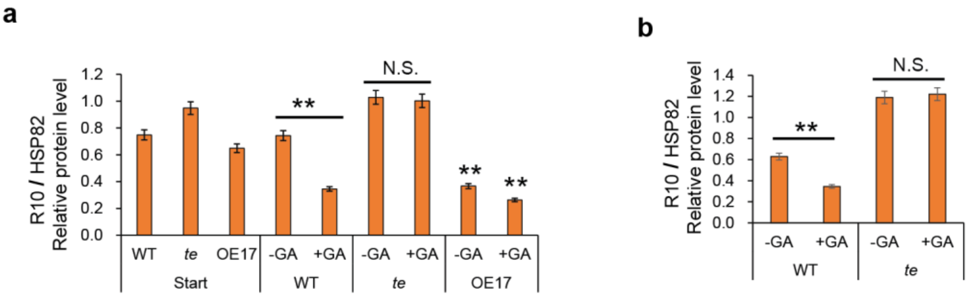

Supplementary Figure 8

The quantification analysis of relative R10/HSP82 protein levels

The quantification analysis of relative R10/HSP82 protein levels corresponding to Figure 4a (**a**) and Figure 4b (**b**). Note: in (**a**), the R10 protein level in OE17 seeds after 4h cycloheximide treatment (regardless of GA application) is notably less than that in OE17 seeds before treatment. Values are means ± s.d. (n=3 replicates). Asterisks mark significant differences according to Student's t test (**P < 0.01). N.S. =no significant.

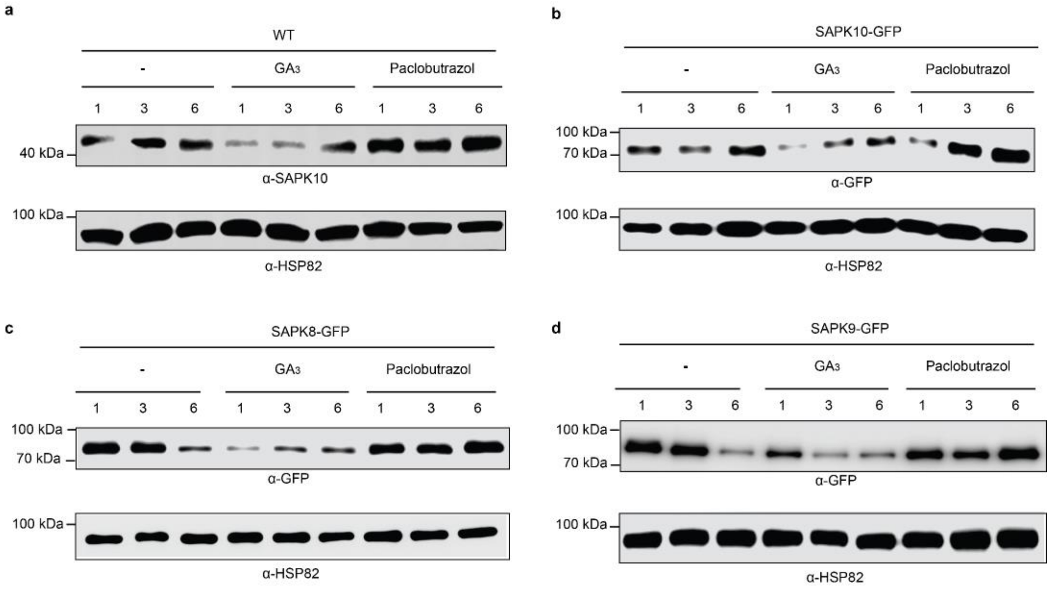

Supplementary Figure 9

Interruption of the GA signaling pathway stabilizes rice SnRK2 proteins.

(**a**) Western blot analysis shows the levels of SAPK10 protein 1, 3 or 6 hours after addition of 100 µM GA_3 or Paclobutrazol (a GA biosynthesis inhibitor). WT: wild type. (**b**-**d**) Western blot analysis shows the levels of SAPK10-GFP, SAPK8-GFP and SAPK9-GFP proteins 1, 3 or 6 hours after addition of 100 µM GA_3 or Paclobutrazol. The 'α-HSP82' signal shows that roughly equal amounts of total plant extracts were used.

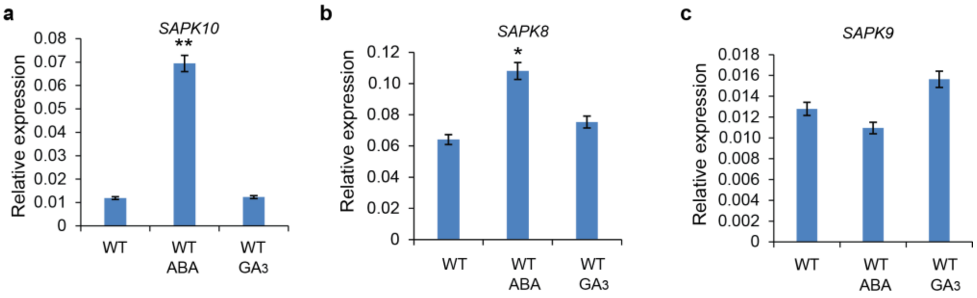

Supplementary Figure 10

Effects of ABA or GA on rice *SnRK2* mRNA expression.

(**a**-**c**) qRT-PCR results show that ABA can promote the expression of *SAPK10* (**a**) and *SAPK8* (**b**) but not *SAPK9* (**c**) in WT plants; but GA_3 does not substantially alter the expression of all three rice *SnRK2* genes (**a**-**c**) in WT plants. Values are means ± s.d. (n=3 replicates). Asterisks mark significant differences according to Student's t test (*P < 0.05, **P < 0.01).

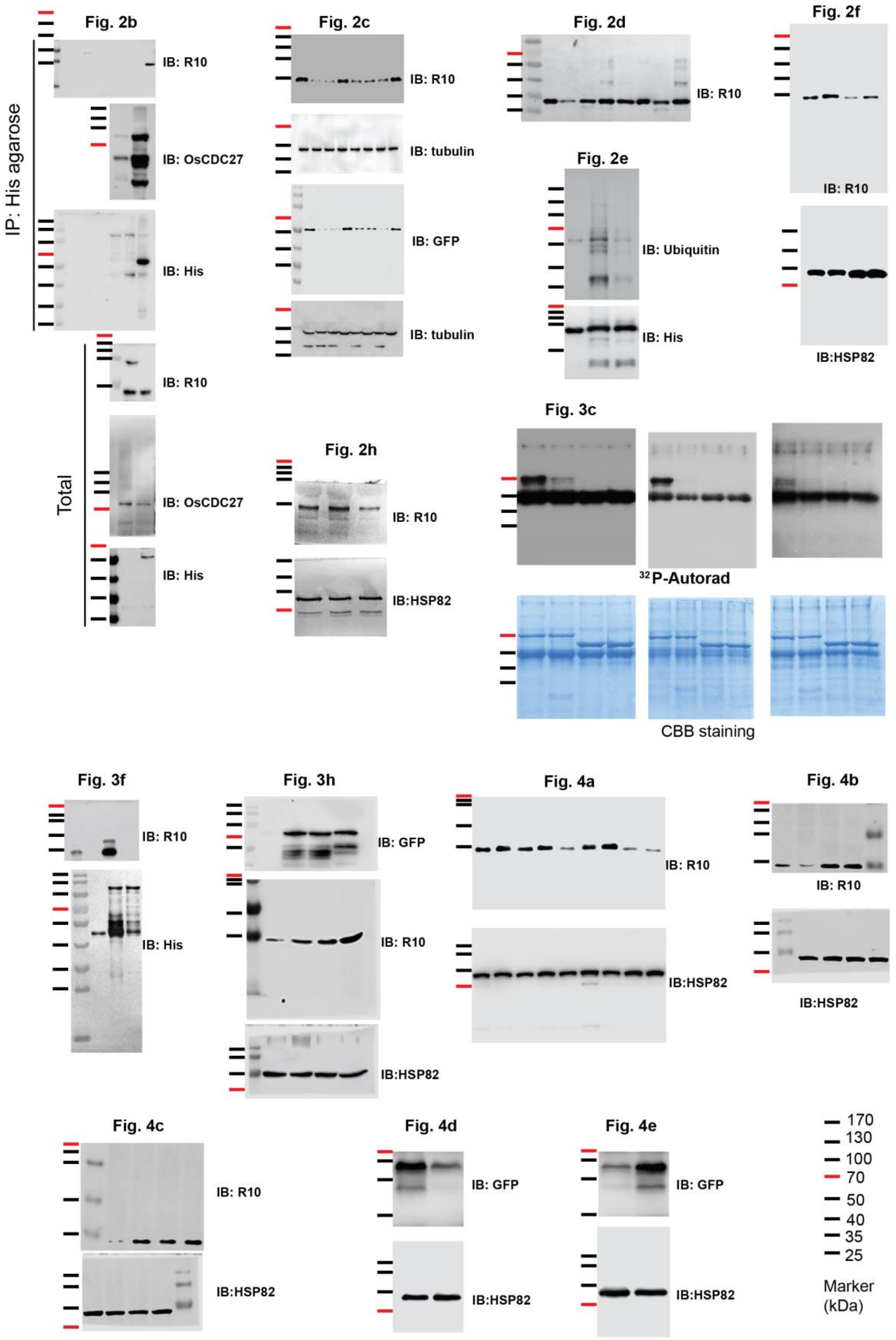

Supplementary Figure 11
Western blot scanned films.

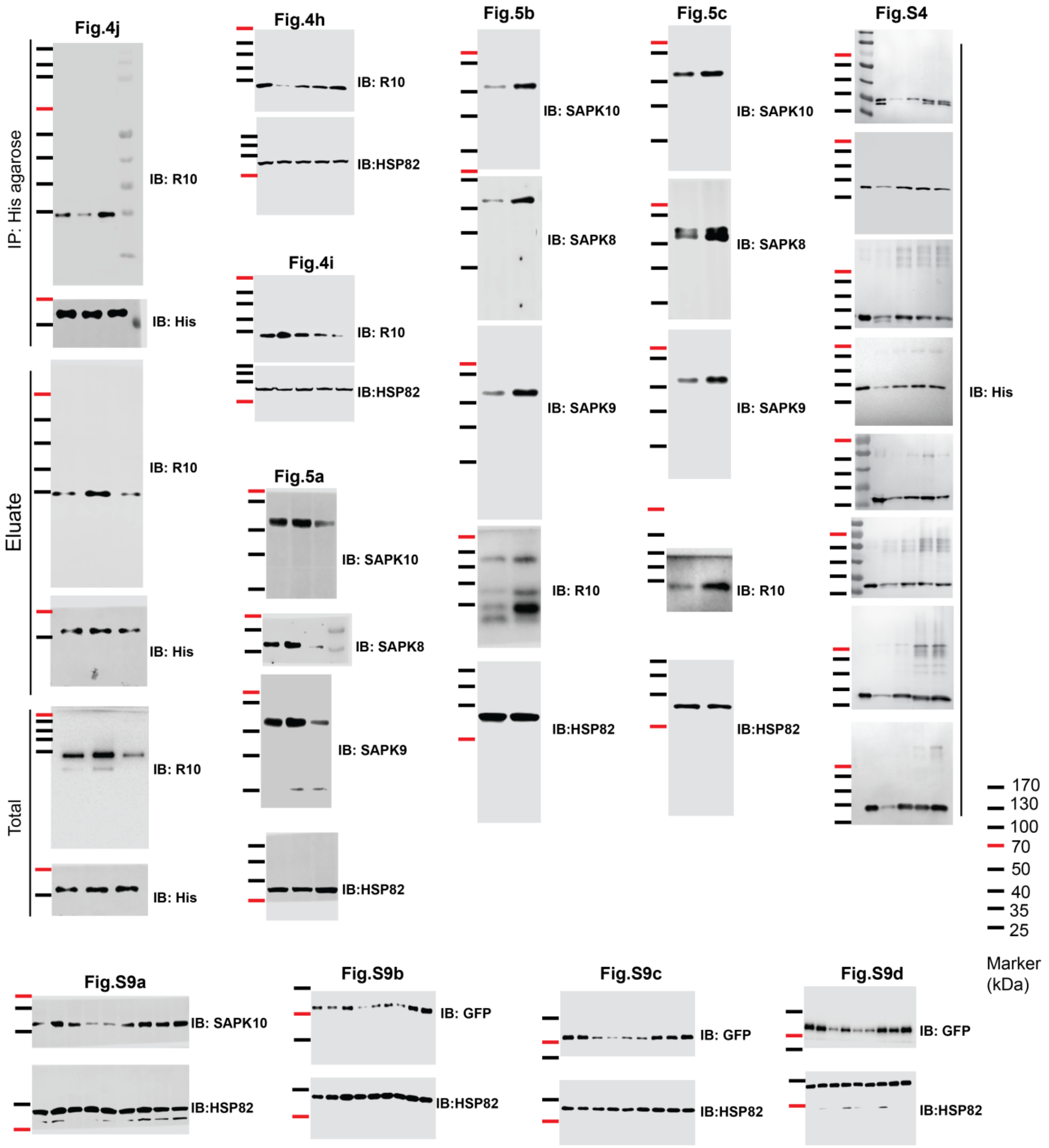

Supplementary Figure 12
Western blot scanned films.

Supplementary Table 1. Primers used for construction of transgenic plasmids

OsPYL/RCAR10 overexpression	RARC10- over- F	TCTGCACTAGGTACCTGCAGATGGAGCAGCAGGAGGAAG
	RARC10- over- R	ATGGATCCGTCGACCTGCAGTTCCGCCGCCGCCGGTGGAG
OsPYL/RCAR10 RNAi	RCAR10-S-F	TTCTGCACTAGGTACCAGGCCTG ATGGAGCAGCAGGAGGAAGTG
	RCAR10-S-R	CTGACGTAGGGGCGATAGAGCTC CTATTCCGCCGCCGCCGGTGGAG
	RCAR10-X-F	CGGGGATCCGTCGACTAC ATGGAGCAGCAGGAGGAAGTG
	RCAR10-X-R	AGGTGGAAGACGCGTTAC CTATTCCGCCGCCGCCGGTGGAG
SAPK Overexpression	SAPK10-over-F	TCTGCACTAGGTACCTGCAGATGGACCGGGCGGCGCTGAC
	SAPK10-over-R	ATGGATCCGTCGACCTGCAG GTGGTGGTGGTGGTGGTGTCACATAGCGTATACTATCTC
	SAPK8-over-F	TCTGCACTAGGTACCTGCAGATGGCAGCGGCGGGGGCCGG
	SAPK8-over-R	ATGGATCCGTCGACCTGCAG GTGGTGGTGGTGGTGGTGTTACATCGCATAGACGATCTC
	SAPK9-over-F	TCTGCACTAGGTACCTGCAGATGGAGAGGGCGGCGGCGGG
	SAPK9-over-R	ATGGATCCGTCGACCTGCAG GTGGTGGTGGTGGTGGTGTTACATGGCATATACGATCTC

Supplementary Table 2. Primers used for construction of protein expression plasmids

**Fragment**	**Forward primer (5’→3’)**	**Reverse primer（5’→3’）**
MBP-TE-His	TTCAGAATTCGGATCCATGGATCACCACCACCACCACCTG	CGACTCTAGAGGATCCTCAATGATGATGATGATGATGATGCCGGATGTAGCTCCTAACAAATGATGT
MBP-TE(S77A)-His	Primer as above	Primer as above, PGEX-4t-1-TE（S77A）plasmid as the PCR template
MBP-TE(S77D)-His	Primer as above	Primer as above, PGEX-4t-1-TE（S77D）plasmid as the PCR template
MBP-TE$_{N195}$-His	AAGGACCATAGCATATATGCAtCAtCTtCCtCCtCCtCCtCCtCGGTCGCCGATGGAGAACT	TAGAGGATCCGAATTCTCAATGATGATGATGATGATGATGGCCAGTGCCCCTCGTCGT
MBP-TE$_{N195}$(S77A)-His	Primer as above	Primer as above, PGEX-4t-1-TE（S77A）plasmid as the PCR template
MBP-TE$_{C155}$-His	TTCAGAATTCGGATCCCAACACTCGGCGCACCCGGTACTG	CGACTCTAGAGGATCCTCAATGATGATGATGATGATGATGCCGGATGTAGCTCCTAACAAATGATGT
MBP-TE$_{C155}$(T457A)-His	Primer as above	Primer as above, PGEX-4t-1-TE（T457A）plasmid as the PCR template
GST-OsPYL/RACAR10	TGGATCCCCGGAATTCATGGAGCAGCAGGAGGAAGTG	GTCGACCCGGGAATTCCTATTCCGCCGCCGCCGGTGGAG
His-OsPYL/RACAR1	TCGCGGATCCGAATTCATGCAACCATCCACCTCTTGC	GACGGAGCTCGAATTCTCATGCATGATCGATCGATCCG
His-OsPYL/RACAR2	TCGCGGATCCGAATTCATGGAGCCCCACATGGAGAG	GACGGAGCTCGAATTCCTAATGGTGGTTGCCGGCGG
His-OsPYL/RACAR3	TCGCGGATCCGAATTCATGGTGGAGGTGGGAGGAGGAGC	GACGGAGCTCGAATTCTCACCGGTCGAGGGGCTCGGTT
His-OsPYL/RACAR4	TCGCGGATCCGAATTCATGCCGTGCATCCCGGCGTCCA	GACGGAGCTCGAATTCTCACGAGCCGGCGGCCCTCGCGC
His-OsPYL/RACAR5	TCGCGGATCCGAATTCATGGTGGGGCTTGTGGGAGGA	GACGGAGCTCGAATTCCTACTGTTCAAGTGGCGAGGTGGGT
His-OsPYL/RACAR6	TCGCGGATCCGAATTCATGCCGTACACCGCTCCACGG	GACGGAGCTCGAATTCCTAGGCGGCGCGCGGCG
His-OsPYL/RACAR7	TCGCGGATCCGAATTCATGAACGGCGCTGGTGGTGC	GACGGAGCTCGAATTCTCAAGGATTGGCAAGGCGCTCCT
His-OsPYL/RACAR8	TCGCGGATCCGAATTCATGAACGGCGTTGGTGGGGC	GACGGAGCTCGAATTCTCAAGGATTGGCAAGGCGCTC
His-OsPYL/RACAR9	TCGCGGATCCGAATTCATGGAGGCGCACGTGGAGAG	GACGGAGCTCGAATTCCTAGTCGCGCCGCCGCGAAGCA
His-OsPYL/RACAR10	TCGCGGATCCGAATTCATGGAGCAGCAGGAGGAAGTG	GACGGAGCTCGAATTCCTATTCCGCCGCCGCCGGTGGAG

D-box mutation in His-OsPYL/RACAR10-m	GAGGCCCTCGACGCCCTCGACGACGC	GGCGTCGAGGGCCTCGGTGCTGGT
His-SAPK10	TCGCGGATCCGAATTCATGGACCGGGCGGCGCTGAC	GACGGAGCTCGAATTCTCACATAGCGTATACTATCTC
His-SAPK8	TCGCGGATCCGAATTCATGGCAGCGGCGGGGGCCGG	GACGGAGCTCGAATTCTTACATCGCATAGACGATCTC
His-SAPK9	TCGCGGATCCGAATTCATGGAGAGGGCGGCGGCGGG	GACGGAGCTCGAATTCTTACATGGCATATACGATCTC
GST-SAPK10	ATTAGAATTCATGGACCGGGCGGCGCTGAC	GCCGCTCGAGTCACATAGCGTATACTATCT
GST-SAPK8(217-361aa)	CCTTTCGAGGATCCTGAAGATCCCAA	TTACATGGCATATACGATCTCTCCGCTGCTCTCAAT
GST-SAPK9(207-371aa)	GCCGGAATTCAAAGAATACGATGGCAAGAC	GCGACTCGAGTTACATCGCATAGACGATCT

Supplementary Table 3. Primers used for construction of BiFC plasmids

Fragment	Forward primer (5’→3’)	Reverse primer（5’→3’）
SAPK8	GCCTACTAGTGGATCC<br>ATGGACCGGGCGGCGCTGAC	CGAGGTCGACGGATCC<br>TCACATAGCGTATACTATCTC
SAPK9	GCCTACTAGTGGATCC<br>ATGGCAGCGGCGGGGGCCGG	CGAGGTCGACGGATCC<br>TTACATCGCATAGACGATCTC
SAPK10	GCCTACTAGTGGATCC<br>ATGGAGAGGGCGGCGGCGGG	CGAGGTCGACGGATCC<br>TTACATGGCATATACGATCTC
R10	CGATAGTACTGTCGAC<br>ATGGAGCAGCAGGAGGAAGTG	TACCCTCGAGGTCGAC<br>TTCCGCCGCCGCCGGTGGAGGAG

Supplementary Table 4. Primers used in Real time Quantitative RT-PCR

**Gene Name**	**Primer sequences（5'→3'）**	
	**Forward primer**	**Reverse primer**
Ubiquitin	ACCACTTCGACCGCCACTACT	ACGCCTAAGCCTGCTGGTT
OsPYL/RCAR1	CTCCAGTCGCTTGCGCGAAC	GATAAGTATGAAAAATAAAGAGCCCG
OsPYL/RCAR3	ACCTGGTTTGGTCTCTGGTGA	GTGCTCATTGTCATCTAACAGCTC
OsPYL/RCAR4	CAAGAACTACCTCTCGGTCACCA	AGGTTGCACTTGACGATGGTGT
OsPYL/RCAR5	GCACATCAAGGCTCCTGTTCAC	GTCTTGACGTTGACCTCGCGC
OsPYL/RCAR6	AGGTTGATGCTTATCGCTTCTTG	TACTCTGTGTTGTCTACAAGCGAAA
OsPYL/RCAR8	AAACCTTTTGTGAGAAACTGTGTAATG	CTCTAACCTCTCAGTGCTCCTTGT
OsPYL/RCAR9	ACACCAGGATGTTCACCGACAC	GACGACGATTTATTGACGAGGC
OsPYL/RCAR10	GCTCGCTCGACATCTTCTTCACTCC	GCCTCCACCGTCGCCCGCA
LEA3	GCCGTGAATGATTTCCCTTTG	CACACCCGTCAGAAATCCTCC
LIP9	TGGAATTTGGAAGTGTTTGGC	CCCACACGAAACACAAACTTC
RAB16A	CATGGACAAGATCAAGGAGAAGC	CTTATTATTCAGGAAGGTGACGTGG
SAPK10	GTGTGGAGTAACCCTCTACGTAATGCT	TGATCTCGGGGATAGAGATTCTAGTG
SAPK8	TGAGATTATAAATCACCGATCGTTGAAA	AGCCTCATCTTCACTGAACCGTACATTC
SAPK9	AGCAACAATTCCAGCAGCAGGCACCA	CTCAATGTCAAGGTCAAGGTCCGAGTCCATA
